# Supplementary material for: Applications of qualitative grounded theory methodology to investigate hearing loss: protocol for a qualitative systematic review
Source: BMJ Open. 2020 Apr 14;10(4):e033537. doi: 10.1136/bmjopen-2019-033537 (PMC7200034; doi:10.1136/bmjopen-2019-033537)
Supplement: Author's manuscript [file bmjopen-2019-033537.draft_revisions.pdf]

BMJ Open

BMJ Open is committed to open peer review. As part of this commitment we make the peer review history of every article we publish publicly available.

When an article is published we post the peer reviewers' comments and the authors' responses online. We also post the versions of the paper that were used during peer review. These are the versions that the peer review comments apply to.

The versions of the paper that follow are the versions that were submitted during the peer review process. They are not the versions of record or the final published versions. They should not be cited or distributed as the published version of this manuscript.

BMJ Open is an open access journal and the full, final, typeset and author-corrected version of record of the manuscript is available on our site with no access controls, subscription charges or pay-per-view fees (http://bmjopen.bmj.com).

If you have any questions on BMJ Open's open peer review process please email info.bmjopen@bmj.com

BMJ Open

Applications of qualitative grounded theory methodology to investigate hearing loss: Protocol for a qualitative systematic review

Journal:	*BMJ Open*
Manuscript ID	bmjopen-2019-033537
Article Type:	Protocol
Date Submitted by the Author:	09-Aug-2019

Complete List of Authors:	Ali, Yasmin; National Institute of Health Research Nottingham Biomedical Research Centre, Hearing Sciences; University of Nottingham, School of Medicine; Clinical Neuroscience; Hearing Sciences<br>Wright, Nicola; University of Nottingham, School of Health Sciences, Queen's Medical Centre<br>Charnock, David; University of Nottingham, School of Health Sciences, Queen's Medical Centre<br>Henshaw, Helen; National Institute of Health Research Nottingham Biomedical Research Centre, Hearing Sciences; The University of Nottingham, National Biomedical Research Centre in Hearing<br>Ferguson, Melanie; Macquarie University, National Acoustic Laboratories, Level 5, Australian Hearing Hub, 16 University Avenue; National Institute of Health Research Nottingham Biomedical Research Centre, Hearing Sciences<br>Hoare, Derek; National Institute of Health Research Nottingham Biomedical Research Centre, Hearing Sciences; University of Nottingham, School of Medicine; Clinical Neuroscience; Hearing Sciences

Keywords:	QUALITATIVE RESEARCH, Hearing Loss, Systematic Review, Grounded Theory

SCHOLARONE™
Manuscripts

I, the Submitting Author has the right to grant and does grant on behalf of all authors of the Work (as defined in the below author licence), an exclusive licence and/or a non-exclusive licence for contributions from authors who are: i) UK Crown employees; ii) where BMJ has agreed a CC-BY licence shall apply, and/or iii) in accordance with the terms applicable for US Federal Government officers or employees acting as part of their official duties; on a worldwide, perpetual, irrevocable, royalty-free basis to BMJ Publishing Group Ltd ("BMJ") its licensees and where the relevant Journal is co-owned by BMJ to the co-owners of the Journal, to publish the Work in this journal and any other BMJ products and to exploit all rights, as set out in our licence.

The Submitting Author accepts and understands that any supply made under these terms is made by BMJ to the Submitting Author unless you are acting as an employee on behalf of your employer or a postgraduate student of an affiliated institution which is paying any applicable article publishing charge ("APC") for Open Access articles. Where the Submitting Author wishes to make the Work available on an Open Access basis (and intends to pay the relevant APC), the terms of reuse of such Open Access shall be governed by a Creative Commons licence – details of these licences and which Creative Commons licence will apply to this Work are set out in our licence referred to above.

Other than as permitted in any relevant BMJ Author's Self Archiving Policies, I confirm this Work has not been accepted for publication elsewhere, is not being considered for publication elsewhere and does not duplicate material already published. I confirm all authors consent to publication of this Work and authorise the granting of this licence.

For peer review only

Applications of qualitative grounded theory methodology to investigate hearing loss: Protocol for a qualitative systematic review

Yasmin H K Ali,[1,2] Nicola Wright,[3] David Charnock,[3] Helen Henshaw,[1,2] Melanie A Ferguson,[1,4] Derek J Hoare[1,2]

[1] National Institute for Health Research (NIHR) Nottingham Biomedical Research Centre, Ropewalk House, 113 The Ropewalk, Nottingham, NG1 5DU, University of Nottingham, Nottingham, UK

[2] Hearing Sciences, Division of Clinical Neuroscience, School of Medicine, University of Nottingham, NG7 2UH, UK

[3] School of Health Sciences, University of Nottingham, Queen's Medical Centre, Nottingham NG7 2HA

[4] National Acoustic Laboratories, Level 5, Australian Hearing Hub, 16 University Avenue, Macquarie University NSW 2109, Australia

Corresponding Author:

Yasmin H. K. Ali

NIHR Nottingham Biomedical Research Centre

113 The Ropewalk

Nottingham, UK

NG1 5DU

Tel: (+44)115 8232627

Email: yasmin.ali@nottingham.ac.uk

ORCID: 0000-0002-0228-6562

Word count: 3,276

Keywords: Hearing loss, grounded theory, qualitative research, systematic review.

For peer review only - http://bmjopen.bmj.com/site/about/guidelines.xhtml

For peer review only

ABTRACT

Introduction: Hearing loss is a chronic condition affecting 11 million individuals in the UK. People with hearing loss regularly experience difficulties interacting in everyday conversations. These difficulties in communication can result in a person with hearing loss withdrawing from social situations and becoming isolated. While hearing health loss research has largely deployed quantitative methods to investigate various aspects of the condition, qualitative research is becoming more widespread. Grounded theory is a specific qualitative methodology that has been used to establish novel theories on the experiences of living with hearing loss.

Method and analysis: The aim of this systematic review is to establish how grounded theory has been applied to investigate the psychosocial aspects of hearing loss. Methods are reported according to the Preferred Reporting Items for Systematic Reviews and Meta-analysis Protocols (PRISMA-P) 2015 checklist. Studies included in this review will have applied grounded theory methodology. For a study to be included, it can apply grounded theory as an overarching methodology, or have grounded theory methodology embedded amongst other methodologies. Studies included will have adult participants (≥18 years) who are either people with an acquired hearing loss, their family and friends (communication partners), or audiologists. The quality of application of grounded theory in each study will be assessed using the Guideline for Reporting and Evaluating Grounded Theory Research Studies (GUREGT).

Ethics and dissemination: As only secondary data will be used in this systematic review, ethical approval is not required. No other ethical issues are foreseen. This review is registered with the International Prospective Register of Systematic Reviews (http://www crd.york.ac.uk/PROSPERO). Findings will be disseminated via peer reviewed publications and at relevant academic conferences. Findings may also be published in relevant professional and third sector newsletters and magazines as appropriate. Data will inform future research and guideline development.

Prospero registration number: PROSPERO CRD42019134

For peer review only

Article summary

Strengths and limitations of this study:

- This systematic review is the first to provide a comprehensive critique of the use of grounded theory to investigate hearing loss.
- The search strategy was formed in collaboration with an information specialist at the University of Nottingham.
- The PRISMA-P guidelines have directed the considerations and layout of this protocol.
- Because experiences and articulations of hearing loss are influenced by age, only adult (≥18 years) participants (people with hearing loss, communication partners, audiologists) will be considered.
- The search will not include grey literature.
- The studies included will only have samples of individuals with hearing loss, rather than full deafness.

For peer review only

INTRODUCTION

Hearing loss is a chronic condition that involves a decrease in an individual's ability to hear sounds. It can occur at mild, moderate, severe, or profound levels, and typically worsens over time[1]. Eleven million people in the UK currently live with hearing loss [2,3]. This number is expected to increase to 15.6 million by 2035[3]. Globally, over 900 million people are expected to acquire a disabling hearing loss by 2050 [2,3].

The annual global societal cost of unaddressed hearing loss is $750 billion[4]. Within the UK, the annual cost of hearing loss is £30 billion, with the larger proportion of this cost dealing with the social, psychological, and health impacts of hearing loss, rather than the treatments provided by audiology services[5]. Untreated hearing loss can impact the work opportunities and occupational progression of people with hearing loss (PHL)[4]. Estimations of the global occupational limitations occurring due to the stigma and inequalities associated with hearing loss is $105 billion annually[4].

PHL experience functional limitations such as communication difficulties in day to day conversations, where speech is often misheard and becomes challenging to follow[6-8]. Failed instances of communication can lead PHL to experience embarrassment, e.g. if incorrect responses are given in a conversation after mishearing others[9,10]. Because PHL struggle to have full interactions with others they can come to feel isolated and separated from the world[10-12]. The anxiousness of repeatedly not being able to hear others can lead to withdrawal from social situations[13]. Consequently, it is estimated that PHL are more likely to develop depression than the general population[14-17].

The impact of hearing loss is not only psychological, as it can also impact interpersonal relationships of PHL also. Not being able to fully communicate and establish successful interactions can also lead to feelings of frustration[18]. Individuals that communicate with PHL, such as their spouses, family and friends, can also become frustrated due to not being heard or understood in interactions[18,19]. Conflict in the relationships of PHL and their communication partners have been reported to occur[18,20,21], with hearing loss being a significant risk factor for the development of abusive relationships[22].

Investigations into the experience of hearing loss are crucial to understanding the implications it has for the person, and the implications it has for their care. To date, most studies in hearing loss research have used quantitative methods. Due to the evident psychosocial impacts of hearing loss, there has been a significant increase in the adoption of qualitative methodologies in hearing loss research[23], particularly since the publication of the "*Conducting qualitative research in audiology: A tutorial*" in 2012[24] . Qualitative methods allow researchers to understand the experiences, opinions and perspectives associated with hearing loss, which experimental measures are not designed to uncover[23-25]. Five main qualitative methodologies exist: phenomenology, ethnography, case studies, narratives, and grounded theory[26]. Each of these methodologies aims to uncover the experiences of a particular sample in different ways, depending on the type of knowledge to be established[26]. Grounded theory is the only qualitative methodology that is exclusively used to establish novel theories completely uninfluenced by previous knowledge[26]. Grounded theory was first established by Glaser and Strauss in 1967[27]. It is defined as the systematic exploration of data in an open-minded, comparative, and rigorous manner for developing a novel theory that is

For peer review only - http://bmjopen.bmj.com/site/about/guidelines.xhtml

purely grounded within the data[28,29]. Through applying grounded theory, people's experiences, and occurring social processes can be understood by integrating definitions and meanings from the perspectives of individuals from the target population[30,31]. To do this, a researcher can adopt the principles of one of three grounded theory schools (Table 1).

Table 1 The three different schools of grounded theory methodology

**Dimensions of Comparison**	**Glaserian School (1967)[27,29]**	**Straussain School (1990)[31]**	**Constructivist School (2006)[28]**
Philosophical stance	Empiricism	Interpretivism	Constructivism
School founders	Barney Glaser & Anselm Strauss	Anselm Strauss & Juliet Corbin	Kathy Charmaz
Philosophical principle	Knowledge is formed based on individual experience, and can be objectively measured.	Knowledge is subjective and socially constructed. Establishing one 'truth' is impossible.	Knowledge is subjective and is constructed by both the participant's experiences and the researcher's own interpretations.
Researcher's influence	Researchers can completely detach themselves from their research and not influence it.	Researchers can't detach themselves from the research and will always influence the research process and findings.	A comprehensive truth is pursued, however it will only be reflective of the social context and group being studied.
Grounded theory emphasis	*Constant comparative analysis*: constantly comparing data and outcomes to establish objective knowledge.	*Reflexivity*: researcher provides reflections on the process of data collection, analysis, and recognises how they influence this process.	*Constant comparative analysis, reflexivity, theoretical sampling* (knowledge is pursued and collected through recruiting different samples and investigating different concepts to develop the theory).

Assessing the methodological applications of grounded theory is essential for determining the trustworthiness and credibility of the theories developed, as suggested by researchers in various fields such as psychology[32], nursing[33]; physiology[34,35] and business & management[36]. A field that has applied and critiqued applications of grounded theory is chronic illness research[37-40]. The founders of all three schools of grounded theory (Table 1) also specialise in investigating chronic illnesses. Glaser & Strauss initially formed the

For peer review only - http://bmjopen.bmj.com/site/about/guidelines.xhtml

methodology to investigate fatal chronic illnesses[27,41], but then used it more extensively to investigate non-fatal chronic conditions[38,42,43]. Charmaz has since identified the methodology as the most appropriate for establishing novel knowledge regarding life-long conditions[28,38,43-45].

Hearing loss is also a chronic disease as recognised by the World Health Organization[46,47], and is the third most common chronic condition affecting the population[48-50]. Despite this none of the aforementioned research into chronic illness reviewing grounded theory included hearing loss as a condition for consideration. Systematic reviews that have investigated hearing loss to date have mainly focused on reviewing phenomena related to hearing loss, such as its impact on interpersonal relationships[18], hearing aids and hearing loss[51], hearing loss and alternative listening devices[52], treatments for PHL with cognitive impairments[53], hearing loss and auditory training[54], vertigo and hearing loss[55], quality of life and hearing loss[56] , and hearing loss and depression[57].

No systematic review investigating hearing loss has yet assessed the use of qualitative methodologies to reach these findings, including grounded theory. The current systematic review will be the first to systematically examine the application of grounded theory methodology to investigate hearing loss. The review aims to (1) describe how grounded theory methodology has been applied within the field of hearing loss, (2) critically appraise the execution of grounded theory methodology in each study, and (3) produce recommendations for future grounded theory methodological applications related to hearing loss.

METHODS AND ANALYSIS

The methods and analysis of this systematic review will be reported in accordance to the Preferred Reporting Items for Systematic Reviews and Meta-Analysis (PRISMA) 2015 checklist[58,59]. The recommended items on the PRISMA-P checklist will form the subheadings of this section. This systematic review will also follow the Cochrane Handbook's suggested approach for conducting methodology-based systematic reviews, i.e. SDMO structure (Studies, Data, Methods, and Outcomes) [60,61]. The SDMO is used to investigate contemporary research methods to illuminate the impact of the methodology on the quality of research within a specific field[60].

For peer review only - http://bmjopen.bmj.com/site/about/guidelines.xhtml

Patient and public involvement

There was no patient and public involvement due to the methodological nature of this study.

ELIGIBILITY CRITERIA

Types of Studies

Studies included in this review will have applied grounded theory methodology. For a study to be included, it can apply grounded theory as an overarching methodology, or be embedded into it amongst other methodologies. These studies can be in the form of retrospective or prospective studies, before and after comparison studies, RCTs, non-RCTs, cohort studies, prospective observational studies, case-control studies, cross-sectional studies, longitudinal studies, or mixed method studies. Purely quantitative studies, studies that have not applied grounded theory methodology, articles reporting expert opinions, case reports, practice guidelines, case series, conference abstracts, and book chapters will be excluded.

Types of Data

Original research submitted to peer-reviewed journals will be the data used in this systematic review. The data collected in those included studies will include qualitative primary data occurring in the form of transcripts or quotes from audio interviews with participants, patient journals, written reflections of patients, family members of PHL, or audiologists, memos of progression of study, initial themes and analysis, and observational. Records reporting diary entries of patients before participation in their prospective study, will also be eligible for inclusion. No secondary data will be collected.

Types of Methods

The methodology under review in this systematic review is qualitative grounded theory methodology. Different methods such as interviews, observations, and focus groups, can all be undertaken using grounded theory methodology. Based on the principles of grounded theory, and the school of grounded theory followed, the most appropriate methods are selected. The methods used in each study will be identified and discussed.

For peer review only - http://bmjopen.bmj.com/site/about/guidelines.xhtml

Types of Outcomes

The explicit use of grounded theory methodology as outlined in the methods section of studies investigating hearing loss. Where available, specific mentions of the applications of grounded theory and the type of school followed will be extracted. Studies must also have researched topics regarding hearing loss with grounded theory methodology, and these topics will also be extracted and collated.

Settings

Any research setting will be included.

Participants

Participants will include PHL with some residual hearing. Studies involving deaf individuals with no residual hearing left will be excluded. This is because being deaf entails significant different experiences to having a hearing loss. Most deaf individuals incorporate being deaf as part of their identity, and learn sign language at an early stage and become a part of the deaf community[62,63]. People with hearing loss on the other hand view their condition as a disability which limits them in their everyday lives, and are less likely to use sign language or be a part of the deaf community as they do not share that identity[64].

Only studies including adults (≥18 years) will be included. The levels of hearing loss for eligibility are mild, moderate, severe, or profound, according to the British Society of Audiology (2018)[65]. Cochlear implant users, hearing aid users, and non-hearing aid users will be included. Studies involving communication partners (friends, family members, and colleagues that interact with PHL) will also be eligible for inclusion. Finally, studies involving audiologists that are involved in hearing loss treatment will be included. Studies with children and young adolescents (<18 years) will be excluded, unless they are in a study with adults where the adult data is reported separately.

Patient and public involvement

For peer review only - http://bmjopen.bmj.com/site/about/guidelines.xhtml

There was no patient and public involvement due to the methodological nature of this study.

Information sources

A systematic search strategy will be employed to identify peer-reviewed journal articles that meet the eligibility criteria. The following databases will be used: PsycINFO (1800S-current), ASSIA (Applied Social Sciences Index and Abstracts, 1987 – current) Global Health (OvidSP database, 1973- current). Web of Science (1899- current) Pubmed (1996-current) British Nursing index (1994- current) CINAHL (Culmative Index to Nursing and Allied Health Literature, 1961- current) MEDLINE (Ovid, In-process & Other Non-Indexed Citations, 1946- current) Scopus (1983- current) EBSCO (1944- current). Google Scholar will be used for forward citation tracking.

Additionally, snowballing of the reference lists of articles shortlisted for inclusion will be undertaken, and related articles from shortlisted authors will be screened in an attempt to identify any relevant articles which may not have been returned by the database searches. The electronic database searches will be updated just before the final analyses and any eligible studies retrieved will be included. At the end of the study selection process, the search strategy produced for each database will be reported, in addition to a PRISMA flow diagram.

Search Strategy

The search strategy was formed from free text and controlled vocabularies (Medical Subject Headings (MeSH)), consultations with an information specialist at the University of Nottingham, and the testing of search results.

1. Hearing Loss, Noise-Induced/ or Hearing Loss/ or Hearing Loss, Sensorineural/ or Hearing Loss, Unilateral/ or Hearing Disorders/ or Hearing Loss, Sudden/ or Hearing Loss, Bilateral/ or Hearing Tests/ or Hearing Loss, Conductive/
2. hearing loss*.mp. or exp Hearing Loss/
3. 1 or 2
4. Amplifiers, Electronic/ or amplifier*.mp.
5. Hearing Aids/ or listening device*.mp.
6. Cochlear Implants/ or cochlear implant*.mp. or Cochlear Implantation/
7. exp Hearing Aids/ or hearing aid*.mp.

For peer review only - http://bmjopen.bmj.com/site/about/guidelines.xhtml

8. 4 or 5 or 6 or 7
9. hard of hearing*.mp.
10. communication partner*.mp.
11. audiologist*.mp. or Audiologists/ or Audiology/
12. exp Persons With Hearing Impairments/
13. 9 or 10 or 11 or 12
14. grounded theory*.mp.
15. exp Grounded Theor*/
16. 14 or 15
17. 3 or 8 or 13
18. 16 and 17

Data management

YA will be responsible for management of the data. A digital record will be kept for all identified articles, and the process of data screening and extraction will be tracked in Covidence. A unique ID code will be assigned to the included articles for access to the full text and data collection sheet.

Selection Process

A review of all titles and abstract resulting from the searches will be performed by YA. During this screening stage, 20% of titles/abstracts will be independently reviewed by a second author. Any differences will be resolved by a third author. Full text screening will be carried out by YA and 20% of full texts will be independently screened by a second author. Any differences between the two authors will be resolved by a third author. Studies that do not meet the inclusion criteria will be excluded from the review and the reason for their exclusion at full text stage will be reported.

Data extraction process

Extracted data will be in the form of direct text collected from the included articles. Data will be stored in Covidence. Prior to starting data extraction guidance notes will be formed by YA.

For peer review only - http://bmjopen.bmj.com/site/about/guidelines.xhtml

For peer review only

Extracted data will include:

1. The study's title, authors, date of publication, number of citations and country;
2. Date(s)/time period of data collection;
3. Aims, objectives, and/or research questions;
4. Participant characteristics: e.g. age, sample size, gender, country and occupation status;
5. Key participant characteristics: type and severity of hearing loss, hearing aid usage, and whether they have a cochlear implantation;
6. Type of participants included (PHL, their family/ friends - communication partners, audiologists, healthcare practitioners);
7. Methods of recruitment of participants;
8. Data collection methods;
9. Particular type of experience of hearing loss being investigated;
10. School of grounded theory followed if mentioned;
11. Ethical standards/ approval;
12. Attempts to establish qualitative rigour or trustworthiness;
13. Study/ methodology limitations;
14. Advantages and disadvantages of using grounded theory if explicated by authors;
15. Study design: methodology and methods;
16. Key findings;
17. Conclusions;
18. Recommendations.

For peer review only

Methodological quality of the individual studies

Quality appraisal of studies will be conducted using the Guideline for Reporting and Evaluating Grounded Theory Research Studies (GUREGT)[66], and critiques of the methodological quality of each individual study will be established.
YA will appraise all records, and 20% of the studies included will be independently appraised by a second author using the same guidelines.

Data synthesis

For peer review only - http://bmjopen.bmj.com/site/about/guidelines.xhtml

A thematic synthesis approach as established by Braun and Clarke will be used to synthesise the findings[67,68]. Through this approach, six steps are undertaken to achieve codes, refine themes, and synthesise the data. The six stages are: data familiarisation, generation of initial codes, searching for themes, reviewing themes, refining and defining themes, and reporting findings. Analysis will firstly consist of coding the data extracted from the included articles and applying initial codes which are close to the analysed data. This will involve establishing which aspects of hearing loss were investigated, how they were investigated, the use of grounded theory, and the grounded theory processes applied within a study. Codes will subsequently be grouped into more focused themes based on the relevance of each concept. These descriptive themes will aid in outlining how grounded theory has been used as a methodology to investigate hearing loss. In the final stage of analysis, analytical codes of the applications of grounded theory and the implications these have for research when investigating hearing loss will be established. Recommendations for how to apply grounded theory when investigating hearing loss will be made.

To ensure inter-rater reliability as required for qualitative rigour[69,70], 20% of included articles will be analysed by a second author. Comparisons of results will be established through meetings and any disputes will be resolved by a third author.

Subgroup analysis

If indicated, studies that are rated as having low quality according to the GUREGT tool will be reviewed separately, to establish the issues that should be resolved when applying grounded theory to investigate hearing loss.

Ethics and dissemination

As only secondary data will be used in this systematic review, no ethical approval is required. No other ethical issues are foreseen. The International Prospective Register of Systematic Reviews (http://www crd.york.ac.uk/PROSPERO) holds the registration record of this systematic review.

Results from the systematic review will be disseminated via peer reviewed publications and relevant academic conferences. Findings may also be published in relevant professional and

For peer review only - http://bmjopen.bmj.com/site/about/guidelines.xhtml

third sector newsletters and magazines as appropriate. Data will be used to inform future research and guideline development.

Outcome of systematic review

A comprehensive understanding of how grounded theory has been used in relation to the study of hearing loss will be identified. Overall, a set of over-arching themes arising from the body of qualitative research that focused on adults' experiences of hearing loss will be established.

Summary

This systematic review will be the first to establish how grounded theory has been used to investigate hearing loss. A critical appraisal of all hearing loss studies that have investigated acquired hearing loss within adult populations using grounded theory will be performed. This novel systematic review will aid in implementing more precise applications of grounded theory in future research. This will also enable more refined understandings of the experiences of hearing loss to be established and facilitate better care for those who have hearing loss.

Contributors

YA is the guarantor of the review (CRD42019134197). YA led on the development of the review protocol and drafted the manuscript. YA, NW, DC, HH and DH contributed to the development of the eligibility criteria, and selection process. NW, DC, HH, and DH all read drafts of the manuscript, provided feedback, and approved the final manuscript.

Exclusive license

The corresponding Author, Yasmin Ali, has the right to grant on behalf of all authors and does grant on behalf of all authors, an exclusive licence (or non-exclusive for government employees) on a worldwide basis to the BMJ Publishing Group Ltd to permit this article (if

For peer review only - http://bmjopen.bmj.com/site/about/guidelines.xhtml

accepted) to be published in BMJ editions and any other BMJPGL products and sublicenses such use and exploit all subsidiary rights, as set out in our licence.

Funding

This systematic review presents independent research with differing sources of funding. YA is funded by the NIHR Nottingham Biomedical Research Programme and Sonova Holding AG. Dr Derek Hoare is funded by the NIHR Biomedical Research Centre Programme. Dr Helen Henshaw is funded through an NIHR Career Development Fellowship (NIHR Ref: CDF-2018-11-ST2-016). Dr Nicola Wright and Dr David Charnock are funded by the University of Nottingham. Dr Melanie Ferguson is funded by the National Acoustic Laboratories in Australia.

Disclaimer

The views expressed are those of the authors and not necessarily those of the NHS, the NIHR, or the Department of Health and Social Care.

Transparency declaration:

Yasmin Ali affirms that the manuscript is an honest, accurate, and transparent account of the study being reported; that no important aspects of the study have been omitted; and that any discrepancies from the study as planned have been explained.

Competing interests

None declared. All authors have completed the Unified Competing Interest form (available on request from the corresponding author) and declare: no exclusive support from any organisation for the submitted work; no financial relationships with any organisations that might have an interest in the submitted work in the previous three years, no other relationships or activities that could appear to have influenced the submitted work.

For peer review only - http://bmjopen.bmj.com/site/about/guidelines.xhtml

Provenance and peer review

Not commissioned; externally peer reviewed.

Data sharing statement

All data collected according to the data items will be available on request if not included in the final published systematic review article.

For peer review only

For peer review only - http://bmjopen.bmj.com/site/about/guidelines.xhtml

References:

1 Alshuaib, W. B., Al-Kandari, J. M. & Hasan, S. M. *Update On Hearing Loss* . InTech (2015).

2 World Health Organization. Addressing the rising prevalence of hearing loss. Report No. 9241550260, (World Health Organization, 2018).

3 Action On Hearing Loss. Hearing Matters Report. (2015). (Available at: https://www.actiononhearingloss.org.uk/-/media/ahl/documents/research-and-policy/reports/hearing-matters-report.pdf

4 World Health Organization. Global costs of unaddressed hearing loss and cost-effectiveness of interventions: a WHO report, 2017. Report No. 9241512040, (World Health Organization, 2017).

5 Archbold, S., Lamb, B., O'Neill, C. & Atkins, J. The real cost of adult hearing loss: reducing its impact by increasing access to the latest hearing technologies. *The Ear Foundation, EarFoundation (Erişim: 12.08. 2017)* (2014).

6 Dobie, R. A., Van Hemel, S. & Council, N. R. Basics of Sound, the Ear, and Hearing. (2004).

7 Dalton, D. S. *et al.* The impact of hearing loss on quality of life in older adults. *Gerontologist* **43**, 661-668 (2003).

8 Knutson, J. F. & Lansing, C. R. The relationship between communication problems and psychological difficulties in persons with profound acquired hearing loss. *Journal of Speech and Hearing Disorders* **55**, 656-664 (1990).

9 Demorest, M. E. & Erdman, S. A. Development of the communication profile for the hearing impaired. *Journal of Speech and Hearing Disorders* **52**, 129-143 (1987).

10 Weinstein, B. E. & Ventry, I. M. Hearing impairment and social isolation in the elderly. *J Speech Hear Res* **25**, 593-599 (1982).

11 Mick, P., Kawachi, I. & Lin, F. R. The Association between Hearing Loss and Social Isolation in Older Adults. *Otolaryng Head Neck* **150**, 378-384, doi:10.1177/0194599813518021 (2014).

12 Heffernan, E., Coulson, N. S. & Ferguson, M. A. Development of the Social Participation Restrictions Questionnaire (SPaRQ) through consultation with adults with hearing loss, researchers, and clinicians: a content evaluation study. *International Journal of Audiology* **57**, 791-799, doi:10.1080/14992027.2018.1483585 (2018).

13 Arlinger, S. Negative consequences of uncorrected hearing loss-a review. *International journal of audiology* **42**, 2S17-12S20 (2003).

14 Gomaa, M. A. M., Elmagd, M. H. A., Elbadry, M. M. & Kader, R. M. A. J. E. A. o. O.-R.-L. Depression, Anxiety and Stress Scale in patients with tinnitus and hearing loss. **271**, 2177-2184 (2014).

15 Kvam, M. H., Loeb, M., Tambs, K. J. T. J. o. d. s. & education, d. Mental health in deaf adults: symptoms of anxiety and depression among hearing and deaf individuals. **12**, 1-7 (2007).

16 Golub, J. S. *et al.* Association of Audiometric Age-Related Hearing Loss With Depressive Symptoms Among Hispanic Individuals. *Jama Otolaryngol* **145**, 132-139, doi:10.1001/jamaoto.2018.3270 (2019).

17 Thomas, A. J. *Acquired hearing loss: Psychological and psychosocial implications*. (Academic Press, 1984).

18 Barker, A. B., Leighton, P. & Ferguson, M. A. Coping together with hearing loss: a qualitative meta-synthesis of the psychosocial experiences of people with hearing loss

For peer review only - http://bmjopen.bmj.com/site/about/guidelines.xhtml

and their communication partners. *International Journal of Audiology* **56**, 297-305, doi:10.1080/14992027.2017.1286695 (2017).
19 Hallberg, L. R. & Barrenas, M. L. Living with a male with noise-induced hearing loss: experiences from the perspective of spouses. *Br J Audiol* **27**, 255-261 (1993).
20 Hetu, R., Jones, L. & Getty, L. The impact of acquired hearing impairment on intimate relationships: implications for rehabilitation. *Audiology* **32**, 363-381 (1993).
21 Heffernan, E., Coulson, N. S., Henshaw, H., Barry, J. G. & Ferguson, M. A. Understanding the psychosocial experiences of adults with mild-moderate hearing loss: An application of Leventhal's self-regulatory model. *International Journal of Audiology* **55**, S3-S12, doi:10.3109/14992027.2015.1117663 (2016).
22 Coker, A. L., Smith, P. H., Bethea, L., King, M. R. & McKeown, R. E. Physical health consequences of physical and psychological intimate partner violence. *Arch Fam Med* **9**, 451-457, doi:DOI 10.1001/archfami.9.5.451 (2000).
23 Bunne, M. Qualitative research methods in otorhinolaryngology. *Int J Pediatr Otorhinolaryngol* **51**, 1-10 (1999).
24 Knudsen, L. V. *et al.* Conducting qualitative research in audiology: A tutorial. **51**, 83-92 (2012).
25 Mugenda, O. M. *Research methods: Quantitative and qualitative approaches*. (African Centre for Technology Studies, 1999).
26 Creswell, J. W. & Poth, C. N. *Qualitative inquiry and research design: Choosing among five approaches*. (Sage publications, 2017).
27 Glaser, B. G., Strauss, A. L. & Strutzel, E. The discovery of grounded theory; strategies for qualitative research. *Nursing research* **17**, 364 (1968).
28 Charmaz, K. *Constructing grounded theory: A practical guide through qualitative analysis*. (Sage, 2006).
29 Glaser, B. G. & Strauss, A. L. *Discovery of grounded theory: Strategies for qualitative research*. (Routledge, 2017).
30 Fassinger, R. E. Paradigms, praxis, problems, and promise: Grounded theory in counseling psychology research. *Journal of counseling psychology* **52**, 156 (2005).
31 Strauss, A. & Corbin, J. M. *Grounded theory in practice*. (Sage, 1997).
32 Weed, M. Research quality considerations for grounded theory research in sport & exercise psychology. *Psychology of sport and exercise* **10**, 502-510 (2009).
33 Lazenbatt, A. & Elliott, N. How to recognise a'quality'grounded theory research study. *Australian Journal of Advanced Nursing, The* **22**, 48 (2005).
34 Ali, N., May, S. & Grafton, K. A systematic review of grounded theory studies in physiotherapy. *Physiotherapy theory and practice*, 1-31 (2018).
35 Hutchison, A. J., Johnston, L. & Breckon, J. Grounded theory-based research within exercise psychology: A critical review. *Qualitative Research in Psychology* **8**, 247-272 (2011).
36 Douglas, D. Grounded theories of management: A methodological review. *Management Research News* **26**, 44-52 (2003).
37 da Silva Barreto, M., Garcia-Vivar, C. & Marcon, S. S. Methodological quality of Grounded Theory research with families living with chronic illness. *International journal of Africa nursing sciences* **8**, 14-22 (2018).
38 Charmaz, K. 'Discovering'chronic illness: using grounded theory. *Soc Sci Med* **30**, 1161-1172 (1990).
39 Baker, C. & Stern, P. N. Finding meaning in chronic illness as the key to self-care. *Canadian Journal of Nursing Research Archive* **25** (1993).
40 Conrad, P. Qualitative research on chronic illness: a commentary on method and conceptual development. *Soc Sci Med* **30**, 1257-1263 (1990).

For peer review only - http://bmjopen.bmj.com/site/about/guidelines.xhtml

41 Glaser, B. G. & Strauss, A. L. *Awareness of dying*. (Routledge, 2017).
42 Corbin, J. & Strauss, A. Managing chronic illness at home: three lines of work. *Qualitative sociology* **8**, 224-247 (1985).
43 Belgrave, L. L. & Charmaz, K. in *The Social Construction of Death* 34-51 (Springer, 2014).
44 Charmaz, K. Loss of self: a fundamental form of suffering in the chronically ill. *Sociology of health & illness* **5**, 168-195 (1983).
45 Charmaz, K. Grounded theory as an emergent method. *Handbook of emergent methods* **155**, 172 (2008).
46 Bernell, S. & Howard, S. W. Use your words carefully: what is a chronic disease? *Frontiers in public health* **4**, 159 (2016).
47 World Health Organization. Preventing chronic diseases: a vital investment. Report No. 9241563001, (World Health Organization, Public Health Agency of Canada, Canada. Public Health Agency of Canada). (2005).
48 Scinicariello, F., Carroll, Y., Eichwald, J., Decker, J. & Breysse, P. N. Association of Obesity with Hearing Impairment in Adolescents. *Sci Rep-Uk* **9**, 1877 (2019).
49 Blackwell, D. L., Lucas, J. W. & Clarke, T. C. Summary health statistics for US adults: national health interview survey, 2012. *Vital and health statistics. Series 10, Data from the National Health Survey*, 1-161 (2014).
50 Vos, T. *et al.* Global, regional, and national incidence, prevalence, and years lived with disability for 310 diseases and injuries, 1990–2015: a systematic analysis for the Global Burden of Disease Study 2015. *The Lancet* **388**, 1545-1602 (2016).
51 Ferguson, M. A. *et al.* Hearing aids for mild to moderate hearing loss in adults. *Cochrane Db Syst Rev*, doi:10.1002/14651858.CD012023.pub2 (2017).
52 Maidment, D. W., Barker, A. B., Xia, J. & Ferguson, M. A. Effectiveness of alternative listening devices to conventional hearing aids for adults with hearing loss: a systematic review protocol. *BMJ open* **6**, e011683 (2016).
53 Mamo, S. K. *et al.* Hearing loss treatment in older adults with cognitive impairment: A systematic review. *Journal of Speech, Language, and Hearing Research* **61**, 2589-2603 (2018).
54 Henshaw, H. & Ferguson, M. A. Efficacy of individual computer-based auditory training for people with hearing loss: A systematic review of the evidence. *PloS one* **8**, e62836 (2013).
55 Yu, H. & Li, H. Association of vertigo with hearing outcomes in patients with sudden sensorineural hearing loss: a systematic review and meta-analysis. *JAMA otolaryngology–head & neck surgery* **144**, 677-683 (2018).
56 Nordvik, Ø. *et al.* Generic quality of life in persons with hearing loss: a systematic literature review. *BMC Ear, Nose and Throat Disorders* **18**, 1 (2018).
57 Lawrence, B. J. *et al.* Hearing loss and depression in older adults: A systematic review and meta-analysis. *The Gerontologist* (2019).
58 Moher, D. *et al.* Preferred reporting items for systematic review and meta-analysis protocols (PRISMA-P) 2015 statement. *Syst Rev* **4**, doi:10.1186/2046-4053-4-1 (2015).
59 Shamseer, L. *et al.* Preferred reporting items for systematic review and meta-analysis protocols (PRISMA-P) 2015: elaboration and explanation. *Bmj-Brit Med J* **349**, doi:10.1136/bmj.g7647 (2015).
60 Munn, Z., Stern, C., Aromataris, E., Lockwood, C. & Jordan, Z. What kind of systematic review should I conduct? A proposed typology and guidance for systematic reviewers in the medical and health sciences. *Bmc Med Res Methodol* **18**, doi:10.1186/s12874-017-0468-4 (2018).

For peer review only - http://bmjopen.bmj.com/site/about/guidelines.xhtml

61 Clarke, M., Oxman, A., Paulsen, E., Higgins, J. & Green, S. Appendix A: Guide to the contents of a Cochrane Methodology protocol and review. *Cochrane Handbook for Systematic Reviews of Interventions* (2009).

62 McIlroy, G. & Storbeck, C. Development of deaf identity: an ethnographic study. *J Deaf Stud Deaf Educ* **16**, 494-511, doi:10.1093/deafed/enr017 (2011).

63 Chapman, M. & Dammeyer, J. The Significance of Deaf Identity for Psychological Well-Being. *J Deaf Stud Deaf Educ*, doi:10.1093/deafed/enw078 (2016).

64 Leigh, I., Marcus, A., Dobosh, P. & Allen, T. Deaf/hearing cultural identity paradigms: modification of the deaf identity development scale. *J Deaf Stud Deaf Educ* **3**, 329-338, doi:10.1093/oxfordjournals.deafed.a014360 (1998).

65 Audiology, B. S. o. Recommended procedure: pure-tone air-conduction and bone-conduction threshold audiometry with and without masking. United Kingdom; (2017).

66 Berthelsen, C. B., Grimshaw-Aagaard, S. L. S. & Hansen, C. Developing a Guideline for Reporting and Evaluating Grounded Theory Research Studies (GUREGT). *International Journal of Health Sciences* **6**, 64-76 (2018).

67 Braun, V. & Clarke, V. What can "thematic analysis" offer health and wellbeing researchers? *Int J Qual Stud Heal* **9**, doi:10.3402/qhw.v9.26152 (2014).

68 Clarke, V. & Braun, V. Thematic analysis. *J Posit Psychol* **12**, 297-298, doi:10.1080/17439760.2016.1262613 (2017).

69 Armstrong, D., Gosling, A., Weinman, J. & Marteau, T. The place of inter-rater reliability in qualitative research: an empirical study. *Sociology* **31**, 597-606 (1997).

70 McAlister, A. *et al.* Qualitative coding: An approach to assess inter-rater reliability. In *ASEE Annual Conference & Exposition, Columbus, Ohio.* https://peer. *asee. org/28777.* (2017).

For peer review only

PRISMA-P (Preferred Reporting Items for Systematic review and Meta-Analysis Protocols) 2015 checklist: recommended items to address in a systematic review protocol*

Section and topic	Item No	Checklist item	Completed? Yes/ No/ N/A
**ADMINISTRATIVE INFORMATION**			
Title:			
Identification	1a	Identify the report as a protocol of a systematic review	Yes
Update	1b	If the protocol is for an update of a previous systematic review, identify as such	Yes
Registration	2	If registered, provide the name of the registry (such as PROSPERO) and registration number	Yes
Authors:			
Contact	3a	Provide name, institutional affiliation, e-mail address of all protocol authors; provide physical mailing address of corresponding author	Yes
Contributions	3b	Describe contributions of protocol authors and identify the guarantor of the review	Yes
Amendments	4	If the protocol represents an amendment of a previously completed or published protocol, identify as such and list changes; otherwise, state plan for documenting important protocol amendments	N/A
Support:			Yes
Sources	5a	Indicate sources of financial or other support for the review	Yes
Sponsor	5b	Provide name for the review funder and/or sponsor	
Role of sponsor or funder	5c	Describe roles of funder(s), sponsor(s), and/or institution(s), if any, in developing the protocol	Yes
**INTRODUCTION**			
Rationale	6	Describe the rationale for the review in the context of what is already known	Yes
Objectives	7	Provide an explicit statement of the question(s) the review will address with reference to participants, interventions, comparators, and outcomes (PICO)	Yes
**METHODS**			
Eligibility criteria	8	Specify the study characteristics (such as PICO, study design, setting,	Yes

For peer review only

		time frame) and report characteristics (such as years considered, language, publication status) to be used as criteria for eligibility for the review	
Information sources	9	Describe all intended information sources (such as electronic databases, contact with study authors, trial registers or other grey literature sources) with planned dates of coverage	Yes
Search strategy	10	Present draft of search strategy to be used for at least one electronic database, including planned limits, such that it could be repeated	Yes
Study records:			
Data management	11a	Describe the mechanism(s) that will be used to manage records and data throughout the review	Yes
Selection process	11b	State the process that will be used for selecting studies (such as two independent reviewers) through each phase of the review (that is, screening, eligibility and inclusion in meta-analysis)	
Data collection process	11c	Describe planned method of extracting data from reports (such as piloting forms, done independently, in duplicate), any processes for obtaining and confirming data from investigators	
Data items	12	List and define all variables for which data will be sought (such as PICO items, funding sources), any pre-planned data assumptions and simplifications	
Outcomes and prioritization	13	List and define all outcomes for which data will be sought, including prioritization of main and additional outcomes, with rationale	Yes
Risk of bias in individual studies	14	Describe anticipated methods for assessing risk of bias of individual studies, including whether this will be done at the outcome or study level, or both; state how this information will be used in data synthesis	Yes
Data synthesis	15a	Describe criteria under which study data will be qualitatively synthesised	N/A
	15b	If data are appropriate for qualitative synthesis, describe planned summary measures, methods of handling data and methods of combining data from studies, including any planned exploration of consistency (such as I^2, Kendall's τ)	N/A
	15c	Describe any proposed additional analyses (such as sensitivity or subgroup analyses, meta-regression)	N/A
	15d	If quantitative synthesis is not appropriate, describe the type of summary planned	Yes
Meta-bias(es)	16	Specify any planned assessment of meta-bias(es) (such as publication	Yes

For peer review only

		bias across studies, selective reporting within studies)	
Confidence in cumulative evidence	17	Describe how the strength of the body of evidence will be assessed (such as GRADE)	Yes

*** It is strongly recommended that this checklist be read in conjunction with the PRISMA-P Explanation and Elaboration (cite when available) for important clarification on the items. Amendments to a review protocol should be tracked and dated. The copyright for PRISMA-P (including checklist) is held by the PRISMA-P Group and is distributed under a Creative Commons Attribution Licence 4.0.**

From: Shamseer L, Moher D, Clarke M, Ghersi D, Liberati A, Petticrew M, Shekelle P, Stewart L, PRISMA-P Group. Preferred reporting items for systematic review and meta-analysis protocols (PRISMA-P) 2015: elaboration and explanation. BMJ. 2015 Jan 2;349(jan02 1):g7647.

For peer review only

BMJ Open

Applications of qualitative grounded theory methodology to investigate hearing loss: Protocol for a qualitative systematic review

Journal:	*BMJ Open*
Manuscript ID	bmjopen-2019-033537.R1
Article Type:	Protocol
Date Submitted by the Author:	02-Dec-2019
Complete List of Authors:	Ali, Yasmin; National Institute of Health Research Nottingham Biomedical Research Centre, Hearing Sciences; University of Nottingham, School of Medicine; Clinical Neuroscience; Hearing Sciences<br>Wright, Nicola; University of Nottingham, School of Health Sciences, Queen's Medical Centre<br>Charnock, David; University of Nottingham, School of Health Sciences, Queen's Medical Centre<br>Henshaw, Helen; National Institute of Health Research Nottingham Biomedical Research Centre, Hearing Sciences; The University of Nottingham, National Biomedical Research Centre in Hearing<br>Hoare, Derek; National Institute of Health Research Nottingham Biomedical Research Centre, Hearing Sciences; University of Nottingham, School of Medicine; Clinical Neuroscience; Hearing Sciences
**Primary Subject Heading**:	Ear, nose and throat/otolaryngology
Secondary Subject Heading:	Research methods, Qualitative research
Keywords:	QUALITATIVE RESEARCH, Hearing Loss, Systematic Review, Grounded Theory

SCHOLARONE™
Manuscripts

I, the Submitting Author has the right to grant and does grant on behalf of all authors of the Work (as defined in the below author licence), an exclusive licence and/or a non-exclusive licence for contributions from authors who are: i) UK Crown employees; ii) where BMJ has agreed a CC-BY licence shall apply, and/or iii) in accordance with the terms applicable for US Federal Government officers or employees acting as part of their official duties; on a worldwide, perpetual, irrevocable, royalty-free basis to BMJ Publishing Group Ltd ("BMJ") its licensees and where the relevant Journal is co-owned by BMJ to the co-owners of the Journal, to publish the Work in this journal and any other BMJ products and to exploit all rights, as set out in our licence.

The Submitting Author accepts and understands that any supply made under these terms is made by BMJ to the Submitting Author unless you are acting as an employee on behalf of your employer or a postgraduate student of an affiliated institution which is paying any applicable article publishing charge ("APC") for Open Access articles. Where the Submitting Author wishes to make the Work available on an Open Access basis (and intends to pay the relevant APC), the terms of reuse of such Open Access shall be governed by a Creative Commons licence – details of these licences and which Creative Commons licence will apply to this Work are set out in our licence referred to above.

Other than as permitted in any relevant BMJ Author's Self Archiving Policies, I confirm this Work has not been accepted for publication elsewhere, is not being considered for publication elsewhere and does not duplicate material already published. I confirm all authors consent to publication of this Work and authorise the granting of this licence.

For peer review only

Applications of qualitative grounded theory methodology to investigate hearing loss: Protocol for a qualitative systematic review

Yasmin H K Ali,[1,2] Nicola Wright,[3] David Charnock,[3] Helen Henshaw,[1,2] Derek J Hoare[1,2]

[1] National Institute for Health Research (NIHR) Nottingham Biomedical Research Centre, Ropewalk House, 113 The Ropewalk, Nottingham, NG1 5DU, University of Nottingham, Nottingham, UK

[2] Hearing Sciences, Division of Clinical Neuroscience, School of Medicine, University of Nottingham, NG7 2UH, UK

[3] School of Health Sciences, University of Nottingham, Queen's Medical Centre, Nottingham NG7 2HA

Corresponding Author:

Yasmin H. K. Ali

NIHR Nottingham Biomedical Research Centre

113 The Ropewalk

Nottingham, UK

NG1 5DU

Tel: (+44)115 8232627

Email: yasmin.ali@nottingham.ac.uk

ORCID: 0000-0002-0228-6562

Word count: 3,840

Keywords: Hearing loss, grounded theory, qualitative research, methodology, systematic review.

For peer review only

ABTRACT

Introduction: Hearing loss is a chronic condition affecting 11 million individuals in the UK. People with hearing loss regularly experience difficulties interacting in everyday conversations. These difficulties in communication can result in a person with hearing loss withdrawing from social situations and becoming isolated. While hearing health loss research has largely deployed quantitative methods to investigate various aspects of the condition, qualitative research is becoming more widespread. Grounded theory is a specific qualitative methodology that has been used to establish novel theories on the experiences of living with hearing loss.

Method and analysis: The aim of this systematic review is to establish how grounded theory has been applied to investigate the psychosocial aspects of hearing loss. Methods are reported according to the Preferred Reporting Items for Systematic Reviews and Meta-analysis Protocols (PRISMA-P) 2015 checklist. Studies included in this review will have applied grounded theory methodology. For a study to be included, it can apply grounded theory as an overarching methodology, or have grounded theory methodology embedded amongst other methodologies. Studies included will have adult participants (≥18 years) who are either people with an acquired hearing loss, their family and friends (communication partners), or audiologists. The quality of application of grounded theory in each study will be assessed using the Guideline for Reporting and Evaluating Grounded Theory Research Studies (GUREGT).

Ethics and dissemination: As only secondary data will be used in this systematic review; ethical approval is not required. No other ethical issues are foreseen. This review is registered with the International Prospective Register of Systematic Reviews (http://www crd.york.ac.uk/PROSPERO). Findings will be disseminated via peer reviewed publications and at relevant academic conferences. Findings may also be published in relevant professional and third sector newsletters and magazines as appropriate. Data will inform future research and guideline development.

For peer review only

Prospero registration number: PROSPERO CRD42019134197

Article summary

Strengths and limitations of this study:

- This systematic review is the first to provide a comprehensive critique of the use of grounded theory to investigate hearing loss.
- Methodological quality of studies is assessed using the Guideline for Reporting and Evaluating Grounded Theory Research Studies (GUREGT).
- Because experiences and articulations of hearing loss are influenced by age, only adult (≥18 years) participants (people with hearing loss, communication partners, audiologists) will be considered.
- The search will not include grey literature.

INTRODUCTION

Hearing loss is a chronic condition that involves a decrease in an individual's ability to hear sounds. It can occur at mild, moderate, severe, or profound levels, and typically worsens over time[1]. Eleven million people in the UK currently live with hearing loss [2,3]. This number is expected to increase to 15.6 million by 2035[3]. Globally, over 900 million people are expected to acquire a disabling hearing loss by 2050 [2,3].

The annual global societal cost of unaddressed hearing loss is $750 billion[4]. Within the UK, the annual cost of hearing loss is £30 billion, with the larger proportion of this cost dealing with the social, psychological, and health impacts of hearing loss, rather than the treatments provided by audiology services[5]. Untreated hearing loss can impact the work opportunities and occupational progression of people with hearing loss (PHL)[4]. Estimations of the global occupational limitations occurring due to the stigma and inequalities associated with hearing loss is $105 billion annually[4].

For peer review only - http://bmjopen.bmj.com/site/about/guidelines.xhtml

PHL experience functional limitations such as communication difficulties in day to day conversations, where speech is often misheard and becomes challenging to follow[6-8]. Failed instances of communication can lead PHL to experience embarrassment, e.g. if incorrect responses are given in a conversation after mishearing others[9,10]. Because PHL struggle to have full interactions with others they can come to feel isolated and separated from the world[10-12]. The anxiousness of repeatedly not being able to hear others can lead to withdrawal from social situations[13]. Consequently, it is estimated that PHL are more likely to develop depression than the general population[14-17].

The impact of hearing loss is not only psychological, as it can also impact interpersonal relationships of PHL also. Not being able to fully communicate and establish successful interactions can also lead to feelings of frustration[18]. Individuals that communicate with PHL, such as their spouses, family and friends, can also become frustrated due to not being heard or understood in interactions[18,19]. Conflict in the relationships of PHL and their communication partners have been reported to occur[18,20,21], with hearing loss being a significant risk factor for the development of abusive relationships[22].

Investigations into the experience of hearing loss are crucial to understanding the implications it has for the person, and the implications it has for their care. To date, most studies in hearing loss research have used quantitative methods. Due to the evident psychosocial impacts of hearing loss, there has been a significant increase in the adoption of qualitative methodologies in hearing loss research[23], particularly since the publication of the "*Conducting qualitative research in audiology: A tutorial"* in 2012 . Qualitative methods allow researchers to understand the experiences, opinions and perspectives associated with hearing loss, which experimental measures are not designed to uncover[23-25].

For peer review only - http://bmjopen.bmj.com/site/about/guidelines.xhtml

There are five main qualitative methodologies; phenomenology, ethnography, and narratives which mainly focus on investigating descriptive characteristics of a phenomenon, and case studies and grounded theory which facilitate both descriptive and explanatory understandings of a phenomenon[26]. Grounded theory is the only qualitative methodology that enables the construction of novel theories that are completely emergent from within the data, and comprehensively explore and explain phenomena[26,27].

Grounded theory was first established by Glaser and Strauss in 1967[28]. It is defined as the systematic exploration of data in an open-minded, comparative, and rigorous manner for developing a novel theory that is purely grounded within the data[27,29]. Through applying grounded theory, people's experiences, and occurring social processes can be understood by integrating definitions and meanings from the perspectives of individuals from the target population[30,31]. To do this, a researcher can adopt the principles of one of three grounded theory schools (Table 1).

Table 1. The three different schools of grounded theory methodology

**Dimensions of Comparison**	**Glaserian School (1967)[28,29]**	**Straussain School (1990)[31]**	**Constructivist School (2006)[27]**
Philosophical stance	Empiricism	Interpretivism	Constructivism
School founders	Barney Glaser & Anselm Strauss	Anselm Strauss & Juliet Corbin	Kathy Charmaz
Philosophical principle	Knowledge is formed based on individual experience and can be objectively measured.	Knowledge is subjective and socially constructed. Establishing one 'truth' is impossible.	Knowledge is subjective and is constructed by both the participant's experiences and the researcher's own interpretations.
Researcher's influence	Researchers can completely detach themselves from their research and not influence it.	Researchers can't detach themselves from the research and will always influence the research process and findings.	A comprehensive truth is pursued; however, it will only be reflective of the social context and group being studied.

For peer review only - http://bmjopen.bmj.com/site/about/guidelines.xhtml

Grounded theory emphasis	*Constant comparative analysis*: constantly comparing data and outcomes to establish objective knowledge.	*Reflexivity*: researcher provides reflections on the process of data collection, analysis, and recognises how they influence this process.	*Constant comparative analysis, reflexivity, theoretical sampling* (knowledge is pursued and collected through recruiting different samples and investigating different concepts to develop the theory).

For peer review only

Assessing the methodological applications of grounded theory is essential for determining the trustworthiness and credibility of the theories developed, as suggested by researchers in various fields such as psychology[32], nursing[33]; physiology[34,35] and business & management[36]. A field that has applied and critiqued applications of grounded theory is chronic illness research[37-40]. The founders of all three schools of grounded theory (Table 1) also specialise in investigating chronic illnesses. Glaser & Strauss initially formed the methodology to investigate fatal chronic illnesses[28,29], but then used it more extensively to investigate non-fatal chronic conditions[38,41,42]. Charmaz has since identified the methodology as the most appropriate for establishing novel knowledge regarding life-long conditions[27,38,42-44]. Hearing loss is also a chronic disease as recognised by the World Health Organization[45,46], and is the third most common chronic condition affecting the population[47-49]. Despite this none of the aforementioned research into chronic illness reviewing grounded theory included hearing loss as a condition for consideration.

There has been an increase in the application of grounded theory methodology in the field of hearing loss[50]. A lack of consistency in the use of grounded theory across healthcare research has been reported[51] despite core principles being maintained across its three schools, and the emphasis of grounded theory being a rigorous systematic process[27,29,50-52]. The literature reinforces the importance of avoiding misinformed applications of grounded theory when investigating hearing loss[24,50], to ensure that theories developed are sufficiently trustworthy for informing subsequent studies[24,50,51,53]. Therefore, this systematic review has outlined grounded

For peer review only - http://bmjopen.bmj.com/site/about/guidelines.xhtml

theory as a qualitative methodology needed for review regarding its applications in hearing loss research.

No methodological systematic review has yet been conducted to assess the quality of studies that use grounded theory to investigate hearing loss. This is essential to inform future applications of grounded theory for improved and trustworthy hearing loss research. Therefore, this review primarily aims to (1) critically appraise the execution of grounded theory methodology in hearing loss research in terms of methodological quality, and (2) produce recommendations for future grounded theory methodological applications pertaining to hearing loss research. A secondary aim is to (3) describe how grounded theory methodology has been applied within the field of hearing loss.

METHODS AND ANALYSIS

The methods and analysis of this systematic review will be reported in accordance to the Preferred Reporting Items for Systematic Reviews and Meta-Analysis (PRISMA) 2015 checklist[54,55]. The recommended items on the PRISMA-P checklist will form the subheadings of this section. This systematic review will also follow the Cochrane Handbook's suggested approach for conducting methodology-based systematic reviews, i.e. SDMO structure (Studies, Data, Methods, and Outcomes) [56,57]. The SDMO is used to investigate contemporary research methods to illuminate the impact of the methodology on the quality of research within a specific field[56].

Timeframe:

Initial searches were conducted in June 2019 but will be updated one month prior to submission of the manuscript for review.

Patient and public involvement

For peer review only - http://bmjopen.bmj.com/site/about/guidelines.xhtml

Patient and public involvement will include the preparation and dissemination of a lay summary of the review findings for a general audience. This will be achieved with appropriate training and support, provided by the NIHR Nottingham Biomedical Research Centre PPI manager.

ELIGIBILITY CRITERIA

Types of Studies

Studies included in this review will have used grounded theory methodology with an appropriate peer-reviewed reference for that adopted approach. For a study to be included, it can apply grounded theory as an overarching methodology, or be embedded into it amongst other qualitative methodologies such as case studies, ethnography, narratives, and phenomenology. During full text screening, if a study explicitly states that grounded theory was used during data collection and/ or analysis, then they will be eligible for inclusion. Studies that do not explicitly state use of grounded theory methodology will be excluded. Included studies can be qualitative or mixed methods research of any design/ methodology*;* retrospective or prospective studies, before and after comparison studies, RCTs, non-RCTs, cohort studies, prospective observational studies, case-control studies, cross-sectional studies, longitudinal studies. Purely quantitative studies, studies that have not applied grounded theory methodology, articles reporting expert opinions, case reports, practice guidelines, case series, conference abstracts, and book chapters will be excluded. Only studies published in English will be included. Any studies published prior to 1967 (when Grounded theory methodology was first introduced to the literature; Glaser and Strauss, 1967[28]) will be excluded.

Types of Data

Original research submitted to peer-reviewed journals will be the data used in this systematic review. The data collected in those included studies will include qualitative primary data occurring in the form of transcripts or quotes from audio interviews with participants, patient journals, written reflections of patients, family members of PHL, or professionals working within audiology, memos of progression of study, initial themes and analyses, and observational notes.

For peer review only - http://bmjopen.bmj.com/site/about/guidelines.xhtml

Records reporting diary entries of patients before participation in their prospective study, will also be eligible for inclusion. No secondary data will be collected.

Types of Methods

The methodology under review in this systematic review is qualitative grounded theory methodology. Different methods such as interviews, observations, and focus groups, can all be undertaken using grounded theory methodology. Based on the principles of grounded theory, and the school of grounded theory followed, the most appropriate methods are selected. The methods used in each study will be identified and discussed.

Types of Outcomes

The outcomes will be extracts taken verbatim from included articles. These extracts will detail the specific steps and decisions made in using grounded theory methodology and how it was applied in the study. Data extracted will include participant characteristics, data collection methods, particular type of hearing loss being investigated, school of grounded theory followed if mentioned, attempts to establish qualitative rigour or trustworthiness, study/ methodology limitations, advantages and disadvantages of using grounded theory if explicated by authors, recommendations, amongst other things. These data will then be critically apprised using the GUREGT guideline for assessing the application of grounded theory methodology in research.

Settings

Any research setting will be included.

Participants

For peer review only - http://bmjopen.bmj.com/site/about/guidelines.xhtml

Participants will include people with mild to profound, acquired or congenital, hearing loss. Studies involving individuals with no residual hearing will be excluded. This is because people with no residual hearing often identify differently (and have very different experiences) to people with some residual hearing[58,59,60]. Studies involving both Deaf and hearing loss participants may ask each individual which group they identify with more and classify them on that basis. These studies will be eligible for inclusion as distinctions between the two groups can be made. Studies that do not specify which participants are Deaf and which have hearing loss will be excluded.

Only studies including adults (≥18 years) will be included. The levels of hearing loss for eligibility are mild, moderate, severe, or profound, according to the British Society of Audiology (2018)[61]. Cochlear implant users, hearing aid users, and non-hearing aid users will be included. Studies involving communication partners (friends, family members, and colleagues that interact with PHL) will also be eligible for inclusion. Finally, studies involving audiologists that are involved in hearing loss treatment will be included. Studies with children and young adolescents (<18 years) will be excluded, unless they are in a study with adults where the adult data is reported separately.

Information sources

A systematic search strategy will be employed to identify peer-reviewed journal articles that meet the eligibility criteria. The following databases will be used: PsycINFO (1800S- current), ASSIA (Applied Social Sciences Index and Abstracts, 1987 – current) Global Health (OvidSP database, 1973- current). Web of Science (1899- current) Pubmed (1996- current) British Nursing index (1994- current) CINAHL (Culmative Index to Nursing and Allied Health Literature, 1961- current) MEDLINE (Ovid, In-process & Other Non-Indexed Citations, 1946- current) Scopus (1983- current) EBSCO (1944- current). Google Scholar will be used for forward citation tracking.

Additionally, snowballing of the reference lists of articles shortlisted for inclusion will be undertaken, and related articles from shortlisted authors will be screened in an attempt to identify any relevant articles which may not have been returned by the database searches. The electronic database searches will be updated just before the final analyses and any eligible studies retrieved

For peer review only - http://bmjopen.bmj.com/site/about/guidelines.xhtml

will be included. At the end of the study selection process, the search strategy produced for each database will be reported, in addition to a PRISMA flow diagram.

To check the feasibility of this systematic review, a scoping search was conducted across multiple databases including Medline, PubMed and PsycINFO. This indicated that there were sufficient relevant papers, approximately 30, to perform a useful synthesis. The searches also informed the key terms for use in the formal search strategy.

Search Strategy

The search strategy was formed from free text and controlled vocabularies (Medical Subject Headings (MeSH)), consultations with an information specialist at the University of Nottingham, and the testing of search results.

1. Hearing Loss, Noise-Induced/ or Hearing Loss/ or Hearing Loss, Sensorineural/ or Hearing Loss, Unilateral/ or Hearing Disorders/ or Hearing Loss, Sudden/ or Hearing Loss, Bilateral/ or Hearing Tests/ or Hearing Loss, Conductive/
2. hearing loss*.mp. or exp Hearing Loss/
3. 1 or 2
4. Amplifiers, Electronic/ or amplifier*.mp.
5. Hearing Aids/ or listening device*.mp.
6. Cochlear Implants/ or cochlear implant*.mp. or Cochlear Implantation/
7. exp Hearing Aids/ or hearing aid*.mp.
8. 4 or 5 or 6 or 7
9. hard of hearing*.mp.
10. communication partner*.mp.
11. audiologist*.mp. or Audiologists/ or Audiology/
12. exp Persons With Hearing Impairments/
13. 9 or 10 or 11 or 12
14. grounded theory*.mp.
15. exp Grounded Theor*/
16. 14 or 15

For peer review only - http://bmjopen.bmj.com/site/about/guidelines.xhtml

17. 3 or 8 or 13
18. 16 and 17

Data management

YA will be responsible for management of the data. A digital record will be kept for all identified articles, and the process of data screening and extraction will be tracked in Covidence. A unique ID code will be assigned to the included articles for access to the full text and data collection sheet.

Selection Process

This systematic review will follow the Centre for Reviews and Dissemination (CRD) guidance for undertaking reviews in healthcare[62] and suggested systematic review practise. All data screening and extraction phases will be independently completed by at least two researchers[62,63]. Any differences will be resolved through discussion or by consulting a third author. Studies that do not meet the inclusion criteria will be excluded from the review and the reason for their exclusion at full text stage will be reported. For a summary of the data selection, extraction and analysis processes, please refer to figure 1.

Data extraction process

After piloting the data extraction form by an independent author, YA will extract data for all studies and this will be checked for accuracy and completeness by a second author, in line with published guidance[62]. Any disagreements that cannot be resolved through discussion will be resolved by a third author. Extracted data will be in the form of direct text collected from the included articles. Data will be stored in Covidence. Prior to starting data extraction guidance notes will be formed by YA.

For peer review only - http://bmjopen.bmj.com/site/about/guidelines.xhtml

Extracted data will include:

1. The study's title, authors, date of publication, number of citations and country;
2. Date(s)/time period of data collection;
3. Aims, objectives, and/or research questions;
4. Source(s) of funding
5. Statement of conflict(s) of interest
6. Participant characteristics: e.g. age, sample size, gender, country and occupation status;
7. Key participant characteristics: type and severity of hearing loss, hearing aid usage, and whether they have a cochlear implantation;
8. Type of participants included (PHL, their family/ friends - communication partners, audiologists, healthcare practitioners);
9. Methods of recruitment of participants;
10. Data collection methods;
11. Particular type of experience of hearing loss being investigated;
12. School of grounded theory followed if mentioned;
13. Ethical standards/ approval;
14. Attempts to establish qualitative rigour or trustworthiness;
15. Study/ methodology limitations;
16. Advantages and disadvantages of using grounded theory if explicated by authors;
17. Study design: methodology and methods;
18. Key findings;
19. Conclusions;
20. Recommendations.

Methodological quality of the individual studies

Quality appraisal of studies will be conducted using the Guideline for Reporting and Evaluating Grounded Theory Research Studies (GUREGT)[64], and critiques of the methodological quality of each individual study will be established. YA will appraise all records, and 100% of the studies

For peer review only

included will be independently appraised by a second author using the same guidelines. A third reviewer will review both critique outcomes and resolve any outstanding disputes.

Data synthesis

A thematic synthesis approach as established by Thomas & Harden[65] will be used to synthesise the findings. Through this approach, four stages will be undertaken to achieve codes, refine themes, and synthesise the data. These four stages are: 1) familiarisation with data through in-depth reading of included papers, 2) line by line coding of the extracted data and critical appraisal results, 3) categorising these codes into descriptive themes, and 4) developing these descriptive themes into analytical ones which provide deeper insights than the original studies[66].

This process will consist of coding the data extracted from the included articles and applying initial codes which are close to the analysed data. This will involve establishing which aspects of hearing loss were investigated, how they were investigated, and how grounded theory was used as a methodology in its applications of central principles such as theoretical sampling, constant comparative analysis and theoretical saturation etc. for investigating the specific hearing loss area. The results of the critical appraisal will then illustrate where grounded theory has been used rigorously and less so, to investigate hearing loss. From descriptive themes, analytical themes will follow, and these will inform a set of guiding statements for researchers wishing to use grounded theory to investigate hearing loss in future studies. Overall, thematic synthesis was chosen as it enables the greater generalisability of results.

The data synthesis will be 100% completed by YA and 100% by a second author, ensuring inter-rater reliability as required for qualitative rigour[67,68]. Results will be compared and discussed in meetings between both authors. A third author will also independently compare both analyses, provide feedback and solve any outstanding disputes. The GRADE-CERQual ('Confidence in the Evidence from Reviews of Qualitative research')[69] tool will be used to assess the confidence of the data Synthesis findings.

For peer review only - http://bmjopen.bmj.com/site/about/guidelines.xhtml

Risk of bias

Within the research team, two are qualitative experts (NW & DC), with the remaining four members having experience of conducting qualitative research investigating aspects of hearing loss (HH, DH, & YA). DC and YA have experience in the application of grounded theory methodologies. To avoid bias during the conduction of this systematic review, each author will reflect on their theoretical predispositions, biases, decisions made during the research process and any problems faced. Risk of bias during analysis will be avoided by assessing studies using the GUREGT guidelines, the GRADE-CERQual tool to assess confidence in the data synthesis, and by considering the funding and conflict(s) of interest statements outlined by each study during data extraction to evaluate whether selective reporting of outcomes was present.

Ethics and dissemination

As only secondary data will be used in this systematic review, no ethical approval is required. No other ethical issues are foreseen. The International Prospective Register of Systematic Reviews (http://www crd.york.ac.uk/PROSPERO) holds the registration record of this systematic review.

Results from the systematic review will be disseminated via peer reviewed publications and relevant academic conferences. Findings may also be published in relevant professional and third sector newsletters and magazines as appropriate. Data will be used to inform future research and guideline development.

Outcome of systematic review

The primary outcome of this review will be a comprehensive understanding of how grounded theory methodolodgy has been used to investigate hearing loss. This will be presented in a detailed table displaying the main themes and subthemes reached after the critical appraisal process using GUREGT and the qualitative thematic synthesis of data. Another primary outcome will be a set of recommendations highlighting the main downfalls of studies that have investigated hearing loss using grounded theory, and guidance for future researchers on how to apply the methodolodgy most effectively. These will be presented in a finalized list based on the

For peer review only - http://bmjopen.bmj.com/site/about/guidelines.xhtml

main themes and subthemes established through critical appraisal and thematic synthesis. A secondary outcome will be a table summarising the topics of hearing loss that were investigated using grounded theory, as established from data extraction.

Summary

This systematic review will be the first to establish how grounded theory has been used to investigate hearing loss. A critical appraisal of all hearing loss studies that have investigated acquired hearing loss within adult populations using grounded theory will be performed. This novel systematic review will aid in implementing more precise applications of grounded theory in future research. This will also enable more refined understandings of the experiences of hearing loss to be established and facilitate better care for those who have hearing loss.

Contributors

YA is the guarantor of the review (CRD42019134197). YA led on the development of the review protocol and drafted the manuscript. YA, NW, DC, HH and DH contributed to the development of the eligibility criteria, and selection process. NW, DC, HH, and DH all read drafts of the manuscript, provided feedback, and approved the final manuscript.

Exclusive license

The corresponding Author, Yasmin Ali, has the right to grant on behalf of all authors and does grant on behalf of all authors, an exclusive licence (or non-exclusive for government employees) on a worldwide basis to the BMJ Publishing Group Ltd to permit this article (if accepted) to be published in BMJ editions and any other BMJPGL products and sublicenses such use and exploit all subsidiary rights, as set out in our licence.

For peer review only - http://bmjopen.bmj.com/site/about/guidelines.xhtml

Funding

This systematic review presents independent research with differing sources of funding. YA is funded by the NIHR Nottingham Biomedical Research Programme and Sonova Holding AG. Dr Derek Hoare is funded by the NIHR Biomedical Research Centre Programme. Dr Helen Henshaw is funded through an NIHR Career Development Fellowship (NIHR Ref: CDF-2018-11-ST2-016). Dr Nicola Wright and Dr David Charnock are funded by the University of Nottingham.

Disclaimer

The views expressed are those of the authors and not necessarily those of the NHS, the NIHR, or the Department of Health and Social Care.

Transparency declaration:

Yasmin Ali affirms that the manuscript is an honest, accurate, and transparent account of the study being reported; that no important aspects of the study have been omitted; and that any discrepancies from the study as planned have been explained.

Competing interests

None declared. All authors have completed the Unified Competing Interest form (available on request from the corresponding author) and declare: no exclusive support from any organisation for the submitted work; no financial relationships with any organisations that might have an interest in the submitted work in the previous three years, no other relationships or activities that could appear to have influenced the submitted work**.** Sonova Holding AG had no input in developing the protocol.

For peer review only

For peer review only - http://bmjopen.bmj.com/site/about/guidelines.xhtml

Provenance and peer review

Not commissioned; externally peer reviewed.

Data sharing statement

All data collected according to the data items will be available on request if not included in the final published systematic review article.

Acknowledgements

Thanks to Dr Melanie Ferguson for comments on an earlier version of this protocol.

For peer review only

References:

1 Alshuaib, W. B., Al-Kandari, J. M. & Hasan, S. M. in *Update On Hearing Loss* (InTech, 2015).

2 World Health Organization. Addressing the rising prevalence of hearing loss. Report No. 9241550260, (World Health Organization, 2018).

3 Action On Hearing Loss. Hearing Matters Report. (2015). (Available at: https://www.actiononhearingloss.org.uk/-/media/ahl/documents/research-and-policy/reports/hearing-matters-report.pdf

4 World Health Organization. Global costs of unaddressed hearing loss and cost-effectiveness of interventions: a WHO report, 2017. Report No. 9241512040, (World Health Organization, 2017).

5 Archbold, S., Lamb, B., O'Neill, C. & Atkins, J. The real cost of adult hearing loss: reducing its impact by increasing access to the latest hearing technologies. *The Ear Foundation, http://www.earfoundation.org.uk/files/download/869 (Erişim: 12.08. 2017)* (2014).

6 Dobie, R. A., Van Hemel, S. & Council, N. R. Basics of Sound, the Ear, and Hearing. (2004).

7 Dalton, D. S. *et al.* The impact of hearing loss on quality of life in older adults. *Gerontologist* **43**, 661-668 (2003).

8 Knutson, J. F. & Lansing, C. R. The relationship between communication problems and psychological difficulties in persons with profound acquired hearing loss. *J. Speech Hear. Disord.* **55**, 656-664 (1990).

9 Demorest, M. E. & Erdman, S. A. Development of the communication profile for the hearing impaired. *J. Speech Hear. Disord.* **52**, 129-143 (1987).

10 Weinstein, B. E. & Ventry, I. M. Hearing impairment and social isolation in the elderly. *J Speech Hear Res* **25**, 593-599 (1982).

11 Mick, P., Kawachi, I. & Lin, F. R. The Association between Hearing Loss and Social Isolation in Older Adults. *Otolaryng Head Neck* **150**, 378-384, doi:10.1177/0194599813518021 (2014).

12 Heffernan, E., Coulson, N. S. & Ferguson, M. A. Development of the Social Participation Restrictions Questionnaire (SPaRQ) through consultation with adults with hearing loss, researchers, and clinicians: a content evaluation study. *International Journal of Audiology* **57**, 791-799, doi:10.1080/14992027.2018.1483585 (2018).

13 Arlinger, S. Negative consequences of uncorrected hearing loss-a review. *International journal of audiology* **42**, 2S17-12S20 (2003).

14 Gomaa, M. A. M., Elmagd, M. H. A., Elbadry, M. M. & Kader, R. M. A. J. E. A. o. O.-R.-L. Depression, Anxiety and Stress Scale in patients with tinnitus and hearing loss. **271**, 2177-2184 (2014).

15 Kvam, M. H., Loeb, M., Tambs, K. J. T. J. o. d. s. & education, d. Mental health in deaf adults: symptoms of anxiety and depression among hearing and deaf individuals. **12**, 1-7 (2007).

For peer review only - http://bmjopen.bmj.com/site/about/guidelines.xhtml

16 Golub, J. S. *et al.* Association of Audiometric Age-Related Hearing Loss With Depressive Symptoms Among Hispanic Individuals. *Jama Otolaryngology-Head & Neck Surgery* **145**, 132-139, doi:10.1001/jamaoto.2018.3270 (2019).
17 Thomas, A. J. *Acquired hearing loss: Psychological and psychosocial implications*. (Academic Press, 1984).
18 Barker, A. B., Leighton, P. & Ferguson, M. A. Coping together with hearing loss: a qualitative meta-synthesis of the psychosocial experiences of people with hearing loss and their communication partners. *International Journal of Audiology* **56**, 297-305, doi:10.1080/14992027.2017.1286695 (2017).
19 Hallberg, L. R. & Barrenas, M. L. Living with a male with noise-induced hearing loss: experiences from the perspective of spouses. *Br J Audiol* **27**, 255-261 (1993).
20 Hetu, R., Jones, L. & Getty, L. The impact of acquired hearing impairment on intimate relationships: implications for rehabilitation. *Audiology* **32**, 363-381 (1993).
21 Heffernan, E., Coulson, N. S., Henshaw, H., Barry, J. G. & Ferguson, M. A. Understanding the psychosocial experiences of adults with mild-moderate hearing loss: An application of Leventhal's self-regulatory model. *International Journal of Audiology* **55**, S3-S12, doi:10.3109/14992027.2015.1117663 (2016).
22 Coker, A. L., Smith, P. H., Bethea, L., King, M. R. & McKeown, R. E. Physical health consequences of physical and psychological intimate partner violence. *Archives of Family Medicine* **9**, 451-457, doi:DOI 10.1001/archfami.9.5.451 (2000).
23 Bunne, M. Qualitative research methods in otorhinolaryngology. *Int J Pediatr Otorhinolaryngol* **51**, 1-10 (1999).
24 Knudsen, L. V. *et al.* Conducting qualitative research in audiology: A tutorial. **51**, 83-92 (2012).
25 Mugenda, O. M. *Research methods: Quantitative and qualitative approaches*. (African Centre for Technology Studies, 1999).
26 Creswell, J. W. & Poth, C. N. *Qualitative inquiry and research design: Choosing among five approaches*. (Sage publications, 2017).
27 Charmaz, K. *Constructing grounded theory: A practical guide through qualitative analysis*. (Sage, 2006).
28 Glaser, B. G., Strauss, A. L. & Strutzel, E. The discovery of grounded theory; strategies for qualitative research. *Nursing research* **17**, 364 (1968).
29 Glaser, B. G. & Strauss, A. L. *Awareness of dying*. (Routledge, 2017).
30 Fassinger, R. E. Paradigms, praxis, problems, and promise: Grounded theory in counseling psychology research. *Journal of counseling psychology* **52**, 156 (2005).
31 Strauss, A. & Corbin, J. M. *Grounded theory in practice*. (Sage, 1997).
32 Weed, M. Research quality considerations for grounded theory research in sport & exercise psychology. *Psychology of sport and exercise* **10**, 502-510 (2009).
33 Lazenbatt, A. & Elliott, N. How to recognise a'quality'grounded theory research study. *Australian Journal of Advanced Nursing, The* **22**, 48 (2005).
34 Ali, N., May, S. & Grafton, K. A systematic review of grounded theory studies in physiotherapy. *Physiotherapy theory and practice*, 1-31 (2018).
35 Hutchison, A. J., Johnston, L. & Breckon, J. Grounded theory-based research within exercise psychology: A critical review. *Qualitative Research in Psychology* **8**, 247-272 (2011).

For peer review only

36 Douglas, D. Grounded theories of management: A methodological review. *Management Research News* **26**, 44-52 (2003).

37 da Silva Barreto, M., Garcia-Vivar, C. & Marcon, S. S. Methodological quality of Grounded Theory research with families living with chronic illness. *International journal of Africa nursing sciences* **8**, 14-22 (2018).

38 Charmaz, K. 'Discovering'chronic illness: using grounded theory. *Social science & medicine* **30**, 1161-1172 (1990).

39 Baker, C. & Stern, P. N. Finding meaning in chronic illness as the key to self-care. *Canadian Journal of Nursing Research Archive* **25** (1993).

40 Conrad, P. Qualitative research on chronic illness: a commentary on method and conceptual development. *Social Science & Medicine* **30**, 1257-1263 (1990).

41 Corbin, J. & Strauss, A. Managing chronic illness at home: three lines of work. *Qualitative sociology* **8**, 224-247 (1985).

42 Belgrave, L. L. & Charmaz, K. in *The Social Construction of Death* 34-51 (Springer, 2014).

43 Charmaz, K. Loss of self: a fundamental form of suffering in the chronically ill. *Sociology of health & illness* **5**, 168-195 (1983).

44 Charmaz, K. Grounded theory as an emergent method. *Handbook of emergent methods* **155**, 172 (2008).

45 Bernell, S. & Howard, S. W. Use your words carefully: what is a chronic disease? *Frontiers in public health* **4**, 159 (2016).

46 World Health Organization. Preventing chronic diseases: a vital investment. Report No. 9241563001, (World Health Organization, Public Health Agency of Canada, Canada. Public Health Agency of Canada, 2005).

47 Scinicariello, F., Carroll, Y., Eichwald, J., Decker, J. & Breysse, P. N. Association of Obesity with Hearing Impairment in Adolescents. *Scientific reports* **9**, 1877 (2019).

48 Blackwell, D. L., Lucas, J. W. & Clarke, T. C. Summary health statistics for US adults: national health interview survey, 2012. *Vital and health statistics. Series 10, Data from the National Health Survey*, 1-161 (2014).

49 Vos, T. *et al.* Global, regional, and national incidence, prevalence, and years lived with disability for 310 diseases and injuries, 1990–2015: a systematic analysis for the Global Burden of Disease Study 2015. *The Lancet* **388**, 1545-1602 (2016).

50 Meston, C. N. & Ng, S. L. A grounded theory primer for audiology. *Seminars in hearing* **33**, 135-146 (2012).

51 Timonen, V., Foley, G. & Conlon, C. Challenges when using grounded theory: A pragmatic introduction to doing GT research. *International Journal of Qualitative Methods* **17**, 1609406918758086 (2018).

52 Morse, J. M. *et al. Developing grounded theory: The second generation*. (Routledge, 2016).

53 Hussein, M. E., Hirst, S., Salyers, V. & Osuji, J. Using grounded theory as a method of inquiry: Advantages and disadvantages. *The Qualitative Report* **19**, 1-15 (2014).

54 Moher, D. *et al.* Preferred reporting items for systematic review and meta-analysis protocols (PRISMA-P) 2015 statement. *Systematic Reviews* **4**, doi:Artn 110.1186/2046-4053-4-1 (2015).

For peer review only - http://bmjopen.bmj.com/site/about/guidelines.xhtml

55 Shamseer, L. *et al.* Preferred reporting items for systematic review and meta-analysis protocols (PRISMA-P) 2015: elaboration and explanation. *Bmj-British Medical Journal* **349**, doi:ARTN g764710.1136/bmj.g7647 (2015).
56 Munn, Z., Stern, C., Aromataris, E., Lockwood, C. & Jordan, Z. What kind of systematic review should I conduct? A proposed typology and guidance for systematic reviewers in the medical and health sciences. *Bmc Medical Research Methodology* **18**, doi:ARTN 510.1186/s12874-017-0468-4 (2018).
57 Clarke, M., Oxman, A., Paulsen, E., Higgins, J. & Green, S. Appendix A: Guide to the contents of a Cochrane Methodology protocol and review. *Cochrane Handbook for Systematic Reviews of Interventions* (2009).
58 McIlroy, G. & Storbeck, C. Development of deaf identity: an ethnographic study. *J Deaf Stud Deaf Educ* **16**, 494-511, doi:10.1093/deafed/enr017 (2011).
59 Chapman, M. & Dammeyer, J. The Significance of Deaf Identity for Psychological Well-Being. *J Deaf Stud Deaf Educ*, doi:10.1093/deafed/enw078 (2016).
60 Leigh, I., Marcus, A., Dobosh, P. & Allen, T. Deaf/hearing cultural identity paradigms: modification of the deaf identity development scale. *J Deaf Stud Deaf Educ* **3**, 329-338, doi:10.1093/oxfordjournals.deafed.a014360 (1998).
61 Audiology, B. S. o. Recommended procedure: pure-tone air-conduction and bone-conduction threshold audiometry with and without masking. . **United Kingdom;** (2017).
62 Reviews, C. f. & Dissemination. *CRD's guidance for undertaking reviews in healthcare*. (York Publ. Services, 2009).
63 Misra, D. P. & Agarwal, V. Systematic Reviews: Challenges for their justification, related comprehensive searches, and implications. *J Korean Med Sci* **33** (2018).
64 Berthelsen, C. B., Grimshaw-Aagaard, S. L. S. & Hansen, C. Developing a Guideline for Reporting and Evaluating Grounded Theory Research Studies (GUREGT). *International Journal of Health Sciences* **6**, 64-76 (2018).
65 Thomas, J. & Harden, A. Methods for the thematic synthesis of qualitative research in systematic reviews. *Bmc Med Res Methodol* **8**, 45 (2008).
66 Ring, N. A., Ritchie, K., Mandava, L. & Jepson, R. A guide to synthesising qualitative research for researchers undertaking health technology assessments and systematic reviews. (2011).
67 Armstrong, D., Gosling, A., Weinman, J. & Marteau, T. The place of inter-rater reliability in qualitative research: an empirical study. *Sociology* **31**, 597-606 (1997).
68 McAlister, A. *et al.* in *ASEE Annual Conference & Exposition, Columbus, Ohio. https://peer.asee.org/28777.*
69 Lewin, S. *et al.* Using Qualitative Evidence in Decision Making for Health and Social Interventions: An Approach to Assess Confidence in Findings from Qualitative Evidence Syntheses (GRADE-CERQual). *Plos Med* **12**, doi:ARTN10.1371/journal.pmed.1001895 (2015).

For peer review only - http://bmjopen.bmj.com/site/about/guidelines.xhtml

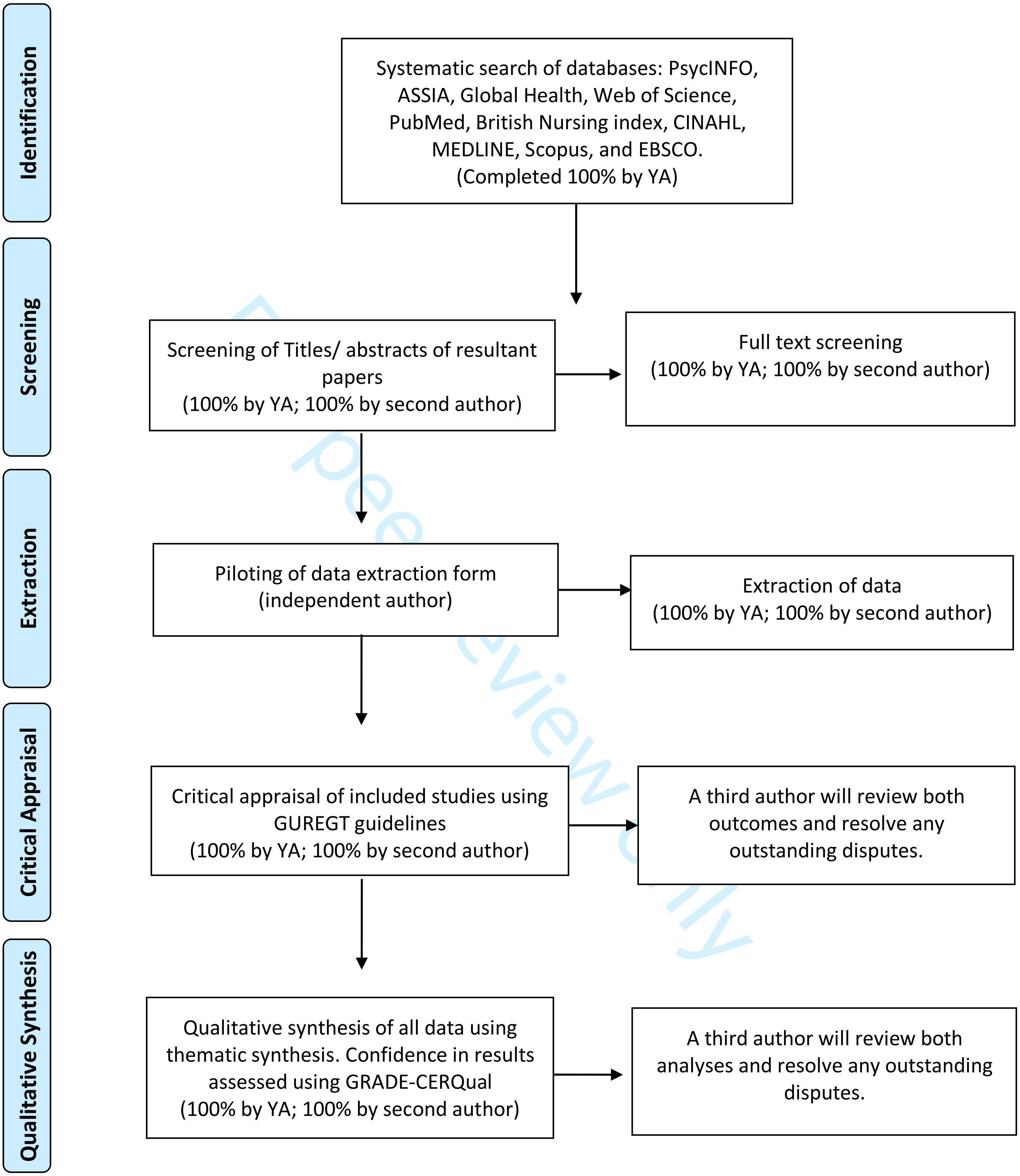

For peer review only

PRISMA-P (Preferred Reporting Items for Systematic review and Meta-Analysis Protocols) 2015 checklist: recommended items to address in a systematic review protocol*

Section and topic	Item No	Checklist item	Completed? Yes/ No/ N/A	Page Numbers
**ADMINISTRATIVE INFORMATION**				
Title:				
Identification	1a	Identify the report as a protocol of a systematic review	Yes	1
Update	1b	If the protocol is for an update of a previous systematic review, identify as such	N/A	N/A
Registration	2	If registered, provide the name of the registry (such as PROSPERO) and registration number	Yes	3
Authors:				
Contact	3a	Provide name, institutional affiliation, e-mail address of all protocol authors; provide physical mailing address of corresponding author	Yes	1
Contributions	3b	Describe contributions of protocol authors and identify the guarantor of the review	Yes	16
Amendments	4	If the protocol represents an amendment of a previously completed or published protocol, identify as such and list changes; otherwise, state plan for documenting important protocol amendments	N/A	N/A
Support:				
Sources	5a	Indicate sources of financial or other support for the review	Yes	17
Sponsor	5b	Provide name for the review funder and/or sponsor	Yes	17
Role of sponsor or funder	5c	Describe roles of funder(s), sponsor(s), and/or institution(s), if any, in developing the protocol	Yes	17-18
**INTRODUCTION**				
Rationale	6	Describe the rationale for the review in the context of what is already known	Yes	3-7
Objectives	7	Provide an explicit statement of the question(s) the review will address with reference to participants, interventions, comparators, and outcomes (PICO)	Yes	7

For peer review only

**METHODS**				
Eligibility criteria	8	Specify the study characteristics (such as PICO, study design, setting, time frame) and report characteristics (such as years considered, language, publication status) to be used as criteria for eligibility for the review	Yes	7-8
Information sources	9	Describe all intended information sources (such as electronic databases, contact with study authors, trial registers or other grey literature sources) with planned dates of coverage	Yes	10-11
Search strategy	10	Present draft of search strategy to be used for at least one electronic database, including planned limits, such that it could be repeated	Yes	11-12
Study records:				
Data management	11a	Describe the mechanism(s) that will be used to manage records and data throughout the review	Yes	12
Selection process	11b	State the process that will be used for selecting studies (such as two independent reviewers) through each phase of the review (that is, screening, eligibility and inclusion in meta-analysis)	Yes	12
Data collection process	11c	Describe planned method of extracting data from reports (such as piloting forms, done independently, in duplicate), any processes for obtaining and confirming data from investigators	Yes	12-14
Data items	12	List and define all variables for which data will be sought (such as PICO items, funding sources), any pre-planned data assumptions and simplifications	Yes	7-14
Outcomes and prioritization	13	List and define all outcomes for which data will be sought, including prioritization of main and additional outcomes, with rationale	Yes	14-17
Risk of bias in individual studies	14	Describe anticipated methods for assessing risk of bias of individual studies, including whether this will be done at the outcome or study level, or both; state how this information will be used in data synthesis	Yes	15-16
Data synthesis	15a	Describe criteria under which study data will be qualitatively synthesised	Yes	14-15
	15b	If data are appropriate for qualitative synthesis, describe planned summary measures, methods of handling data and methods of combining data from studies, including any planned exploration of consistency (such as I^2, Kendall's τ)	Yes	14- 16
	15c	Describe any proposed additional analyses (such as sensitivity or subgroup analyses, meta-regression)	N/A	N/A

For peer review only

	15d	If quantitative synthesis is not appropriate, describe the type of summary planned	Yes	14-16
Meta-bias(es)	16	Specify any planned assessment of meta-bias(es) (such as publication bias across studies, selective reporting within studies)	Yes	15-16
Confidence in cumulative evidence	17	Describe how the strength of the body of evidence will be assessed (such as GRADE)	Yes	15-16

*** It is strongly recommended that this checklist be read in conjunction with the PRISMA-P Explanation and Elaboration (cite when available) for important clarification on the items. Amendments to a review protocol should be tracked and dated. The copyright for PRISMA-P (including checklist) is held by the PRISMA-P Group and is distributed under a Creative Commons Attribution Licence 4.0.**

From: Shamseer L, Moher D, Clarke M, Ghersi D, Liberati A, Petticrew M, Shekelle P, Stewart L, PRISMA-P Group. Preferred reporting items for systematic review and meta-analysis protocols (PRISMA-P) 2015: elaboration and explanation. BMJ. 2015 Jan 2;349(jan02 1):g7647.

For peer review only

BMJ Open

Applications of qualitative grounded theory methodology to investigate hearing loss: Protocol for a qualitative systematic review

Journal:	*BMJ Open*
Manuscript ID	bmjopen-2019-033537.R2
Article Type:	Protocol
Date Submitted by the Author:	04-Feb-2020

Complete List of Authors:	Ali, Yasmin; National Institute of Health Research Nottingham Biomedical Research Centre, Hearing Sciences; University of Nottingham, School of Medicine; Clinical Neuroscience; Hearing Sciences<br>Wright, Nicola; University of Nottingham, School of Health Sciences, Queen's Medical Centre<br>Charnock, David; University of Nottingham, School of Health Sciences, Queen's Medical Centre<br>Henshaw, Helen; National Institute of Health Research Nottingham Biomedical Research Centre, Hearing Sciences; The University of Nottingham, National Biomedical Research Centre in Hearing<br>Hoare, Derek; National Institute of Health Research Nottingham Biomedical Research Centre, Hearing Sciences; University of Nottingham, School of Medicine; Clinical Neuroscience; Hearing Sciences

**Primary Subject Heading**:	Ear, nose and throat/otolaryngology
Secondary Subject Heading:	Research methods, Qualitative research
Keywords:	QUALITATIVE RESEARCH, Hearing Loss, Systematic Review, Grounded Theory

SCHOLARONE™
Manuscripts

I, the Submitting Author has the right to grant and does grant on behalf of all authors of the Work (as defined in the below author licence), an exclusive licence and/or a non-exclusive licence for contributions from authors who are: i) UK Crown employees; ii) where BMJ has agreed a CC-BY licence shall apply, and/or iii) in accordance with the terms applicable for US Federal Government officers or employees acting as part of their official duties; on a worldwide, perpetual, irrevocable, royalty-free basis to BMJ Publishing Group Ltd ("BMJ") its licensees and where the relevant Journal is co-owned by BMJ to the co-owners of the Journal, to publish the Work in this journal and any other BMJ products and to exploit all rights, as set out in our licence.

The Submitting Author accepts and understands that any supply made under these terms is made by BMJ to the Submitting Author unless you are acting as an employee on behalf of your employer or a postgraduate student of an affiliated institution which is paying any applicable article publishing charge ("APC") for Open Access articles. Where the Submitting Author wishes to make the Work available on an Open Access basis (and intends to pay the relevant APC), the terms of reuse of such Open Access shall be governed by a Creative Commons licence – details of these licences and which Creative Commons licence will apply to this Work are set out in our licence referred to above.

Other than as permitted in any relevant BMJ Author's Self Archiving Policies, I confirm this Work has not been accepted for publication elsewhere, is not being considered for publication elsewhere and does not duplicate material already published. I confirm all authors consent to publication of this Work and authorise the granting of this licence.

For peer review only

Applications of qualitative grounded theory methodology to investigate hearing loss: Protocol for a qualitative systematic review

Yasmin H K Ali,[1,2] Nicola Wright,[3] David Charnock,[3] Helen Henshaw,[1,2] Derek J Hoare[1,2]

[1] National Institute for Health Research (NIHR) Nottingham Biomedical Research Centre, Ropewalk House, 113 The Ropewalk, Nottingham, NG1 5DU, University of Nottingham, Nottingham, UK

[2] Hearing Sciences, Division of Clinical Neuroscience, School of Medicine, University of Nottingham, NG7 2UH, UK

[3] School of Health Sciences, University of Nottingham, Queen's Medical Centre, Nottingham NG7 2HA

Corresponding Author:

Yasmin H. K. Ali

NIHR Nottingham Biomedical Research Centre

113 The Ropewalk

Nottingham, UK

NG1 5DU

Tel: (+44)115 8232627

Email: yasmin.ali@nottingham.ac.uk

ORCID: 0000-0002-0228-6562

Word count: **3,879**

Keywords: Hearing loss, grounded theory, qualitative research, methodology, systematic review.

For peer review only

ABTRACT

Introduction: Hearing loss is a chronic condition affecting 11 million individuals in the UK. People with hearing loss regularly experience difficulties interacting in everyday conversations. These difficulties in communication can result in a person with hearing loss withdrawing from social situations and becoming isolated. While hearing health loss research has largely deployed quantitative methods to investigate various aspects of the condition, qualitative research is becoming more widespread. Grounded theory is a specific qualitative methodology that has been used to establish novel theories on the experiences of living with hearing loss.

Method and analysis: The aim of this systematic review is to establish how grounded theory has been applied to investigate the psychosocial aspects of hearing loss. Methods are reported according to the Preferred Reporting Items for Systematic Reviews and Meta-analysis Protocols (PRISMA-P) 2015 checklist. Studies included in this review will have applied grounded theory as an overarching methodology or have grounded theory embedded amongst other methodologies. Studies included will have adult participants (≥18 years) who are either people with an acquired hearing loss, their family and friends (communication partners), or healthcare practitioners including audiologists, GPs, ENT specialists, and hearing therapists. The quality of application of grounded theory in each study will be assessed using the Guideline for Reporting and Evaluating Grounded Theory Research Studies (GUREGT).

Ethics and dissemination: As only secondary data will be used in this systematic review; ethical approval is not required. No other ethical issues are foreseen. This review is registered with the International Prospective Register of Systematic Reviews (http://www crd.york.ac.uk/PROSPERO). Findings will be disseminated via peer reviewed publications and at relevant academic conferences. Findings may also be published in relevant professional and third sector newsletters and magazines as appropriate. Data will inform future research and guideline development.

For peer review only

Prospero registration number: PROSPERO CRD42019134197

Article summary

Strengths and limitations of this study:

- This systematic review is the first to provide a comprehensive critique of the use of grounded theory to investigate hearing loss.
- Methodological quality of studies is assessed using the Guideline for Reporting and Evaluating Grounded Theory Research Studies (GUREGT).
- Because experiences and articulations of hearing loss are influenced by age, only adult (≥18 years) participants (people with hearing loss, communication partners, and healthcare practitioners) will be considered.
- The search will not include grey literature.

INTRODUCTION

Hearing loss is a chronic condition that involves a decrease in an individual's ability to hear sounds. It can occur at mild, moderate, severe, or profound levels, and typically worsens over time[1]. Eleven million people in the UK currently live with hearing loss [2,3]. This number is expected to increase to 15.6 million by 2035[3]. Globally, over 900 million people are expected to acquire a disabling hearing loss by 2050 [2,3].

The annual global societal cost of unaddressed hearing loss is $750 billion[4]. Within the UK, the annual cost of hearing loss is £30 billion, with the larger proportion of this cost dealing with the social, psychological, and health impacts of hearing loss, rather than the treatments provided by audiology services[5]. Untreated hearing loss can impact the work opportunities and occupational progression of people with hearing loss (PHL)[4]. Estimations of the global occupational limitations occurring due to the stigma and inequalities associated with hearing loss is $105 billion annually[4].

For peer review only - http://bmjopen.bmj.com/site/about/guidelines.xhtml

PHL experience functional limitations such as communication difficulties in day to day conversations, where speech is often misheard and becomes challenging to follow[6-8]. Failed instances of communication can lead PHL to experience embarrassment, e.g. if incorrect responses are given in a conversation after mishearing others[9,10]. Because PHL struggle to have full interactions with others they can come to feel isolated and separated from the world[10-12]. The anxiousness of repeatedly not being able to hear others can lead to withdrawal from social situations[13]. Consequently, it is estimated that PHL are more likely to develop depression than the general population[14-17].

The impact of hearing loss is not only psychological, as it can impact interpersonal relationships of PHL also. Not being able to fully communicate and establish successful interactions can also lead to feelings of frustration[18]. Individuals that communicate with PHL, such as their spouses, family and friends, can also become frustrated due to not being heard or understood in interactions[18,19]. Conflict in the relationships of PHL and their communication partners have been reported to occur[18,20,21], with hearing loss being a significant risk factor for the development of abusive relationships[22].

Investigations into the experience of hearing loss are crucial to understanding the implications it has for the person, and the implications it has for their care. To date, most studies in hearing loss research have used quantitative methods. Due to the evident psychosocial impacts of hearing loss, there has been a significant increase in the adoption of qualitative methodologies in hearing loss research[23], particularly since the publication of the "*Conducting qualitative research in audiology: A tutorial*" in 2012 . Qualitative methods allow researchers to understand the experiences, opinions and perspectives associated with hearing loss, which experimental measures are not designed to uncover[23-25].

For peer review only - http://bmjopen.bmj.com/site/about/guidelines.xhtml

There are five main qualitative methodologies; phenomenology, ethnography, and narratives which mainly focus on investigating descriptive characteristics of a phenomenon, and case studies and grounded theory which facilitate both descriptive and explanatory understandings of a phenomenon[26]. Grounded theory is a qualitative methodology developed specifically to enable the construction of novel theories that are directly emergent from within the data, which comprehensively explore and explain phenomena[26,27].

Grounded theory was first established by Glaser and Strauss in 1967[28]. It is defined as the systematic exploration of data in an open-minded, comparative, and rigorous manner for developing a novel theory that is purely grounded within the data[27,29]. Through applying grounded theory, people's experiences, and occurring social processes can be understood by integrating definitions and meanings from the perspectives of individuals from the target population[30,31]. To do this, a researcher can adopt the principles of one of three grounded theory schools (Table 1).

Table 1. The three different schools of grounded theory methodology

**Dimensions of Comparison**	**Glaserian School (1967)[28,29]**	**Straussain School (1990)[31]**	**Constructivist School (2006)[27]**
Philosophical stance	Empiricism	Interpretivism	Constructivism
School founders	Barney Glaser & Anselm Strauss	Anselm Strauss & Juliet Corbin	Kathy Charmaz
Philosophical principle	Knowledge is formed based on individual experience and can be objectively measured.	Knowledge is subjective and socially constructed. Establishing one 'truth' is impossible.	Knowledge is subjective and is constructed by both the participant's experiences and the researcher's own interpretations.
Researcher's influence	Researchers can completely detach themselves from their research and not influence it.	Researchers can't detach themselves from the research and will always influence the research process and findings.	A comprehensive truth is pursued; however, it will only be reflective of the social context and group being studied.

For peer review only - http://bmjopen.bmj.com/site/about/guidelines.xhtml

Grounded theory emphasis	*Constant comparative analysis*: constantly comparing data and outcomes to establish objective knowledge.	*Reflexivity*: researcher provides reflections on the process of data collection, analysis, and recognises how they influence this process.	*Constant comparative analysis, reflexivity, theoretical sampling* (knowledge is pursued and collected through recruiting different samples and investigating different concepts to develop the theory).

Assessing the methodological applications of grounded theory is essential for determining the trustworthiness and credibility of the theories developed, as suggested by researchers in various fields such as psychology[32], nursing[33]; physiology[34,35] and business & management[36]. A field that has applied and critiqued applications of grounded theory is chronic illness research[37-40]. The founders of all three schools of grounded theory (Table 1) also specialise in investigating chronic illnesses. Glaser & Strauss initially formed the methodology to investigate fatal chronic illnesses[28,29], but then used it more extensively to investigate non-fatal chronic conditions[38,41,42]. Charmaz has since identified the methodology as the most appropriate for establishing novel knowledge regarding life-long conditions[27,38,42-44]. Hearing loss is also a chronic disease as recognised by the World Health Organization[45,46], and is the third most common chronic condition affecting the population[47-49]. Despite this none of the aforementioned research into chronic illness reviewing grounded theory included hearing loss as a condition for consideration.

There has been an increase in the application of grounded theory methodology in the field of hearing loss[50]. A lack of consistency in the use of grounded theory across healthcare research has been reported[51] despite core principles being maintained across its three schools, and the emphasis of grounded theory being a rigorous systematic process[27,29,50-52]. The literature reinforces the importance of avoiding misinformed applications of grounded theory when investigating hearing loss[24,50], to ensure that theories developed are sufficiently trustworthy for informing subsequent studies[24,50,51,53]. Therefore, this systematic review has outlined grounded

For peer review only

For peer review only - http://bmjopen.bmj.com/site/about/guidelines.xhtml

theory as a qualitative methodology needed for review regarding its applications in hearing loss research.

No methodological systematic review has yet been conducted to assess the quality of studies that use grounded theory to investigate hearing loss. This is essential to inform future applications of grounded theory for improved and trustworthy hearing loss research. Therefore, this review primarily aims to (1) critically appraise the execution of grounded theory methodology in hearing loss research in terms of methodological quality, and (2) produce recommendations for future grounded theory methodological applications pertaining to hearing loss research. A secondary aim is to (3) describe how grounded theory methodology has been applied within the field of hearing loss.

METHODS AND ANALYSIS

The methods and analysis of this systematic review will be reported in accordance to the Preferred Reporting Items for Systematic Reviews and Meta-Analysis (PRISMA) 2015 checklist[54,55]. The recommended items on the PRISMA-P checklist will form the subheadings of this section. This systematic review will also follow the Cochrane Handbook's suggested approach for conducting methodology-based systematic reviews, i.e. SDMO structure (Studies, Data, Methods, and Outcomes) [56,57]. The SDMO is used to investigate contemporary research methods to illuminate the impact of the methodology on the quality of research within a specific field[56].

Timeframe:

Initial searches were conducted in June 2019 but will be updated one month prior to submission of the manuscript for review.

Patient and public involvement

For peer review only - http://bmjopen.bmj.com/site/about/guidelines.xhtml

People with hearing loss were consulted in the identification of the most relevant healthcare practitioner roles when seeking hearing healthcare services. The prominent professions identified were audiologists, GPs, ENT specialists, and hearing therapists. Therefore, they have been included within the inclusion criteria.

Patient and public involvement will also include the preparation and dissemination of a lay summary of the review findings for a general audience. This will be achieved with appropriate training and support, provided by the NIHR Nottingham Biomedical Research Centre PPI manager.

For peer review only

ELIGIBILITY CRITERIA

Types of Studies

Studies included in this review will have used grounded theory methodology with an appropriate peer-reviewed reference for that adopted approach. For a study to be included, it can apply grounded theory as an overarching methodology, or be embedded into it amongst other qualitative methodologies such as case studies, ethnography, narratives, and phenomenology. During full text screening, if a study explicitly states that grounded theory was used during data collection and/ or analysis, then they will be eligible for inclusion. Studies that do not explicitly state use of grounded theory methodology will be excluded. Purely quantitative studies, studies that have not applied grounded theory methodology, articles reporting expert opinions, case reports, practice guidelines, case series, conference abstracts, and book chapters will be excluded. Only studies published in English will be included. Any studies published prior to 1967 (when Grounded theory methodology was first introduced to the literature; Glaser and Strauss, 1967[28]) will be excluded.

Types of Data

Original research submitted to peer-reviewed journals will be the data used in this systematic review. The data collected in those included studies will include qualitative primary data occurring in the form of transcripts or quotes from audio interviews with participants, patient

journals, written reflections of patients, family members of PHL, or professionals working within audiology, memos of progression of study, initial themes and analyses, and observational notes. Records reporting diary entries of patients before participation in their prospective study, will also be eligible for inclusion. No secondary data will be collected.

Types of Methods

The methodology under review in this systematic review is qualitative grounded theory methodology. Different methods such as interviews, observations, and focus groups, can all be undertaken using grounded theory methodology. Based on the principles of grounded theory, and the school of grounded theory followed, the most appropriate methods are selected. The methods used in each study will be identified and discussed.

Types of Outcomes

The outcomes will be extracts taken verbatim from included articles. These extracts will detail the specific steps and decisions made in using grounded theory methodology and how it was applied in the study. Data extracted will include participant characteristics, data collection methods, particular type of hearing loss being investigated, school of grounded theory followed if mentioned, attempts to establish qualitative rigour or trustworthiness, study/ methodology limitations, advantages and disadvantages of using grounded theory if explicated by authors, recommendations, amongst other things. These data will then be critically apprised using the GUREGT guideline for assessing the application of grounded theory methodology in research.

Settings

Any research setting will be included.

Participants

For peer review only - http://bmjopen.bmj.com/site/about/guidelines.xhtml

Participants will include people with mild to profound, acquired or congenital, hearing loss. Studies involving individuals with no residual hearing will be excluded. This is because people with no residual hearing often identify differently (and have very different experiences) to people with some residual hearing[58,59,60]. Studies involving both Deaf and hearing loss participants may ask each individual which group they identify with more and classify them on that basis. These studies will be eligible for inclusion as distinctions between the two groups can be made. Studies that do not specify which participants are Deaf and which have hearing loss will be excluded.

Only studies including adults (≥18 years) will be included. The levels of hearing loss for eligibility are mild, moderate, severe, or profound, according to the British Society of Audiology (2018)[61]. Cochlear implant users, hearing aid users, and non-hearing aid users will be included. Studies involving communication partners (friends, family members, and colleagues that interact with PHL) will also be eligible for inclusion. Studies including healthcare practitioners such as audiologists, GPs (general practitioners), ENT specialists (ear, nose and throat), and hearing therapists involved in hearing loss treatment will be considered. Studies with children and young adolescents (<18 years) will be excluded, unless they are in a study with adults where the adult data is reported separately.

Information sources

A systematic search strategy will be employed to identify peer-reviewed journal articles that meet the eligibility criteria. The following databases will be used: PsycINFO (1800S- current), ASSIA (Applied Social Sciences Index and Abstracts, 1987 – current) Global Health (OvidSP database, 1973- current). Web of Science (1899- current) Pubmed (1996- current) British Nursing index (1994- current) CINAHL (Culmative Index to Nursing and Allied Health Literature, 1961- current) MEDLINE (Ovid, In-process & Other Non-Indexed Citations, 1946- current) Scopus (1983- current) EBSCO (1944- current). Google Scholar will be used for forward citation tracking.

Additionally, snowballing of the reference lists of articles shortlisted for inclusion will be undertaken, and related articles from shortlisted authors will be screened in an attempt to identify any relevant articles which may not have been returned by the database searches. The electronic

For peer review only

For peer review only - http://bmjopen.bmj.com/site/about/guidelines.xhtml

database searches will be updated just before the final analyses and any eligible studies retrieved will be included. At the end of the study selection process, the search strategy produced for each database will be reported, in addition to a PRISMA flow diagram.

To check the feasibility of this systematic review, a scoping search was conducted across multiple databases including Medline, PubMed and PsycINFO. This indicated that there were sufficient relevant papers, approximately 30, to perform a useful synthesis. The searches also informed the key terms for use in the formal search strategy.

For peer review only

Search Strategy

The search strategy was formed from free text and controlled vocabularies (Medical Subject Headings (MeSH)), consultations with an information specialist at the University of Nottingham, and the testing of search results.

1. Hearing Loss, Noise-Induced/ or Hearing Loss/ or Hearing Loss, Sensorineural/ or Hearing Loss, Unilateral/ or Hearing Disorders/ or Hearing Loss, Sudden/ or Hearing Loss, Bilateral/ or Hearing Tests/ or Hearing Loss, Conductive/
2. hearing loss*.mp. or exp Hearing Loss/
3. 1 or 2
4. Amplifiers, Electronic/ or amplifier*.mp.
5. Hearing Aids/ or listening device*.mp.
6. Cochlear Implants/ or cochlear implant*.mp. or Cochlear Implantation/
7. exp Hearing Aids/ or hearing aid*.mp.
8. 4 or 5 or 6 or 7
9. hard of hearing*.mp.
10. communication partner*.mp.
11. audiologist*.mp. or Audiologists/ or Audiology/
12. exp Persons With Hearing Impairments/
13. 9 or 10 or 11 or 12
14. grounded theory*.mp.
15. exp Grounded Theor*/

For peer review only - http://bmjopen.bmj.com/site/about/guidelines.xhtml

16. 14 or 15
17. 3 or 8 or 13
18. 16 and 17

Data management

YA will be responsible for management of the data. A digital record will be kept for all identified articles, and the process of data screening and extraction will be tracked in Covidence. A unique ID code will be assigned to the included articles for access to the full text and data collection sheet.

Selection Process

This systematic review will follow the Centre for Reviews and Dissemination (CRD) guidance for undertaking reviews in healthcare[62] and suggested systematic review practise. All data screening and extraction phases will be independently completed by at least two researchers[62,63]. Any differences will be resolved through discussion or by consulting a third author. Studies that do not meet the inclusion criteria will be excluded from the review and the reason for their exclusion at full text stage will be reported. For a summary of the data selection, extraction and analysis processes, please refer to figure 1.

Data extraction process

After piloting the data extraction form by an independent author, YA will extract data for all studies and 100% of the studies will be extracted by a second researcher(s) in line with published guidance[62]. Any disagreements that cannot be resolved through discussion will be resolved by a third author. Extracted data will be in the form of direct text collected from the included articles. Data will be stored in Covidence. Prior to starting data extraction guidance notes will be formed by YA.

For peer review only - http://bmjopen.bmj.com/site/about/guidelines.xhtml

For peer review only

Extracted data will include:

1. The study's title, authors, date of publication, number of citations and country;
2. Date(s)/time period of data collection;
3. Aims, objectives, and/or research questions;
4. Source(s) of funding
5. Statement of conflict(s) of interest
6. Participant characteristics: e.g. age, sample size, gender, country and occupation status;
7. Key participant characteristics: type and severity of hearing loss, hearing aid usage, and whether they have a cochlear implantation;
8. Type of participants included (PHL, their family/ friends - communication partners, healthcare practitioners such as audiologists, GPs, ENT specialists, hearing therapists);
9. Methods of recruitment of participants;
10. Data collection methods;
11. Particular type of experience of hearing loss being investigated;
12. School of grounded theory followed if mentioned;
13. Ethical standards/ approval;
14. Attempts to establish qualitative rigour or trustworthiness;
15. Study/ methodology limitations;
16. Advantages and disadvantages of using grounded theory if explicated by authors;
17. Study design: methodology and methods;
18. Key findings;
19. Conclusions;
20. Recommendations.

Methodological quality of the individual studies

Quality appraisal of studies will be conducted using the Guideline for Reporting and Evaluating Grounded Theory Research Studies (GUREGT)[64], and critiques of the methodological quality of each individual study will be established. YA will appraise all records, and 100% of the studies

For peer review only - http://bmjopen.bmj.com/site/about/guidelines.xhtml

included will be independently appraised by a second author using the same guidelines. A third reviewer will review both critique outcomes and resolve any outstanding disputes.

Data synthesis

A thematic synthesis approach as established by Thomas & Harden[65] will be used to synthesise the findings. Through this approach, four stages will be undertaken to achieve codes, refine themes, and synthesise the data. These four stages are: 1) familiarisation with data through in-depth reading of included papers, 2) line by line coding of the extracted data and critical appraisal results, 3) categorising these codes into descriptive themes, and 4) developing these descriptive themes into analytical ones which provide deeper insights than the original studies[66].

This process will consist of coding the data extracted from the included articles and applying initial codes which are close to the analysed data. This will involve establishing which aspects of hearing loss were investigated, how they were investigated, and how grounded theory was used as a methodology in its applications of central principles such as theoretical sampling, constant comparative analysis and theoretical saturation etc. for investigating the specific hearing loss area. The results of the critical appraisal will then illustrate where grounded theory has been used rigorously and less so, to investigate hearing loss. From descriptive themes, analytical themes will follow, and these will inform a set of guiding statements for researchers wishing to use grounded theory to investigate hearing loss in future studies. Overall, thematic synthesis was chosen as it enables the greater generalisability of results.

The data synthesis will be 100% completed by YA and 100% by a second author, ensuring inter-rater reliability as required for qualitative rigour[67,68]. Results will be compared and discussed in meetings between both authors. A third author will also independently compare both analyses, provide feedback and solve any outstanding disputes. The GRADE-CERQual (‘Confidence in the Evidence from Reviews of Qualitative research’)[69] tool will be used to assess the confidence of the data Synthesis findings.

For peer review only - http://bmjopen.bmj.com/site/about/guidelines.xhtml

Risk of bias

Within the research team, two are qualitative experts (NW & DC), with the remaining four members having experience of conducting qualitative research investigating aspects of hearing loss (HH, DH, & YA). DC and YA have experience in the application of grounded theory methodologies. To avoid bias during the conduction of this systematic review, each author will reflect on their theoretical predispositions, biases, decisions made during the research process and any problems faced. Risk of bias during analysis will be avoided by assessing studies using the GUREGT guidelines, the GRADE-CERQual tool to assess confidence in the data synthesis, and by considering the funding and conflict(s) of interest statements outlined by each study during data extraction to evaluate whether selective reporting of outcomes was present.

Ethics and dissemination

As only secondary data will be used in this systematic review, no ethical approval is required. No other ethical issues are foreseen. The International Prospective Register of Systematic Reviews (http://www crd.york.ac.uk/PROSPERO) holds the registration record of this systematic review.

Results from the systematic review will be disseminated via peer reviewed publications and relevant academic conferences. Findings may also be published in relevant professional and third sector newsletters and magazines as appropriate. Data will be used to inform future research and guideline development.

Outcome of systematic review

The primary outcome of this review will be a comprehensive understanding of how grounded theory methodolodgy has been used to investigate hearing loss. This will be presented in a detailed table displaying the main themes and subthemes reached after the critical appraisal process using GUREGT and the qualitative thematic synthesis of data. Another primary outcome will be a set of recommendations highlighting the main downfalls of studies that have investigated hearing loss using grounded theory, and guidance for future researchers on how to apply the methodolodgy most effectively. These will be presented in a finalized list based on the

For peer review only - http://bmjopen.bmj.com/site/about/guidelines.xhtml

main themes and subthemes established through critical appraisal and thematic synthesis. A secondary outcome will be a table summarising the topics of hearing loss that were investigated using grounded theory, as established from data extraction.

Summary

This systematic review will be the first to establish how grounded theory has been used to investigate hearing loss. A critical appraisal of all hearing loss studies that have investigated acquired hearing loss within adult populations using grounded theory will be performed. This novel systematic review will aid in implementing more precise applications of grounded theory in future research. This will also enable more refined understandings of the experiences of hearing loss to be established and facilitate better care for those who have hearing loss.

Contributors

YA is the guarantor of the review (CRD42019134197). YA led on the development of the review protocol and drafted the manuscript. YA, NW, DC, HH and DH contributed to the development of the eligibility criteria, and selection process. NW, DC, HH, and DH all read drafts of the manuscript, provided feedback, and approved the final manuscript.

Exclusive license

The corresponding Author, Yasmin Ali, has the right to grant on behalf of all authors and does grant on behalf of all authors, an exclusive licence (or non-exclusive for government employees) on a worldwide basis to the BMJ Publishing Group Ltd to permit this article (if accepted) to be published in BMJ editions and any other BMJPGL products and sublicenses such use and exploit all subsidiary rights, as set out in our licence.

For peer review only - http://bmjopen.bmj.com/site/about/guidelines.xhtml

Funding

This systematic review presents independent research with differing sources of funding. YA is funded by the NIHR Nottingham Biomedical Research Programme and Sonova Holding AG. Dr Derek Hoare is funded by the NIHR Biomedical Research Centre Programme. Dr Helen Henshaw is funded through an NIHR Career Development Fellowship (NIHR Ref: CDF-2018-11-ST2-016). Dr Nicola Wright and Dr David Charnock are funded by the University of Nottingham.

Disclaimer

The views expressed are those of the authors and not necessarily those of the NHS, the NIHR, or the Department of Health and Social Care.

Transparency declaration:

Yasmin Ali affirms that the manuscript is an honest, accurate, and transparent account of the study being reported; that no important aspects of the study have been omitted; and that any discrepancies from the study as planned have been explained.

Competing interests

None declared. All authors have completed the Unified Competing Interest form (available on request from the corresponding author) and declare: no exclusive support from any organisation for the submitted work; no financial relationships with any organisations that might have an interest in the submitted work in the previous three years, no other relationships or activities that could appear to have influenced the submitted work**.** Sonova Holding AG had no input in developing the protocol.

For peer review only

Provenance and peer review

Not commissioned; externally peer reviewed.

Data sharing statement

All data collected according to the data items will be available on request if not included in the final published systematic review article.

Acknowledgements

Thanks to Dr Melanie Ferguson for comments on an earlier version of this protocol.

Figure Legend:

Figure 1. Flowchart outlining the systematic review process.

For peer review only

For peer review only - http://bmjopen.bmj.com/site/about/guidelines.xhtml

References:

1 Alshuaib, W. B., Al-Kandari, J. M. & Hasan, S. M. in *Update On Hearing Loss* (InTech, 2015).
2 World Health Organization. Addressing the rising prevalence of hearing loss. Report No. 9241550260, (World Health Organization, 2018).
3 Action On Hearing Loss. Hearing Matters Report. (2015). (Available at: https://www.actiononhearingloss.org.uk/-/media/ahl/documents/research-and-policy/reports/hearing-matters-report.pdf
4 World Health Organization. Global costs of unaddressed hearing loss and cost-effectiveness of interventions: a WHO report, 2017. Report No. 9241512040, (World Health Organization, 2017).
5 Archbold, S., Lamb, B., O'Neill, C. & Atkins, J. The real cost of adult hearing loss: reducing its impact by increasing access to the latest hearing technologies. *The Ear Foundation, http://www.earfoundation.org.uk/files/download/869 (Erişim: 12.08. 2017)* (2014).
6 Dobie, R. A., Van Hemel, S. & Council, N. R. Basics of Sound, the Ear, and Hearing. (2004).
7 Dalton, D. S. *et al.* The impact of hearing loss on quality of life in older adults. *Gerontologist* **43**, 661-668 (2003).
8 Knutson, J. F. & Lansing, C. R. The relationship between communication problems and psychological difficulties in persons with profound acquired hearing loss. *J. Speech Hear. Disord.* **55**, 656-664 (1990).
9 Demorest, M. E. & Erdman, S. A. Development of the communication profile for the hearing impaired. *J. Speech Hear. Disord.* **52**, 129-143 (1987).
10 Weinstein, B. E. & Ventry, I. M. Hearing impairment and social isolation in the elderly. *J Speech Hear Res* **25**, 593-599 (1982).
11 Mick, P., Kawachi, I. & Lin, F. R. The Association between Hearing Loss and Social Isolation in Older Adults. *Otolaryng Head Neck* **150**, 378-384, doi:10.1177/0194599813518021 (2014).
12 Heffernan, E., Coulson, N. S. & Ferguson, M. A. Development of the Social Participation Restrictions Questionnaire (SPaRQ) through consultation with adults with hearing loss, researchers, and clinicians: a content evaluation study. *International Journal of Audiology* **57**, 791-799, doi:10.1080/14992027.2018.1483585 (2018).
13 Arlinger, S. Negative consequences of uncorrected hearing loss-a review. *International journal of audiology* **42**, 2S17-12S20 (2003).
14 Gomaa, M. A. M., Elmagd, M. H. A., Elbadry, M. M. & Kader, R. M. A. J. E. A. o. O.-R.-L. Depression, Anxiety and Stress Scale in patients with tinnitus and hearing loss. **271**, 2177-2184 (2014).
15 Kvam, M. H., Loeb, M., Tambs, K. J. T. J. o. d. s. & education, d. Mental health in deaf adults: symptoms of anxiety and depression among hearing and deaf individuals. **12**, 1-7 (2007).

For peer review only - http://bmjopen.bmj.com/site/about/guidelines.xhtml

16 Golub, J. S. *et al.* Association of Audiometric Age-Related Hearing Loss With Depressive Symptoms Among Hispanic Individuals. *Jama Otolaryngology-Head & Neck Surgery* **145**, 132-139, doi:10.1001/jamaoto.2018.3270 (2019).
17 Thomas, A. J. *Acquired hearing loss: Psychological and psychosocial implications*. (Academic Press, 1984).
18 Barker, A. B., Leighton, P. & Ferguson, M. A. Coping together with hearing loss: a qualitative meta-synthesis of the psychosocial experiences of people with hearing loss and their communication partners. *International Journal of Audiology* **56**, 297-305, doi:10.1080/14992027.2017.1286695 (2017).
19 Hallberg, L. R. & Barrenas, M. L. Living with a male with noise-induced hearing loss: experiences from the perspective of spouses. *Br J Audiol* **27**, 255-261 (1993).
20 Hetu, R., Jones, L. & Getty, L. The impact of acquired hearing impairment on intimate relationships: implications for rehabilitation. *Audiology* **32**, 363-381 (1993).
21 Heffernan, E., Coulson, N. S., Henshaw, H., Barry, J. G. & Ferguson, M. A. Understanding the psychosocial experiences of adults with mild-moderate hearing loss: An application of Leventhal's self-regulatory model. *International Journal of Audiology* **55**, S3-S12, doi:10.3109/14992027.2015.1117663 (2016).
22 Coker, A. L., Smith, P. H., Bethea, L., King, M. R. & McKeown, R. E. Physical health consequences of physical and psychological intimate partner violence. *Archives of Family Medicine* **9**, 451-457, doi:DOI 10.1001/archfami.9.5.451 (2000).
23 Bunne, M. Qualitative research methods in otorhinolaryngology. *Int J Pediatr Otorhinolaryngol* **51**, 1-10 (1999).
24 Knudsen, L. V. *et al.* Conducting qualitative research in audiology: A tutorial. **51**, 83-92 (2012).
25 Mugenda, O. M. *Research methods: Quantitative and qualitative approaches*. (African Centre for Technology Studies, 1999).
26 Creswell, J. W. & Poth, C. N. *Qualitative inquiry and research design: Choosing among five approaches*. (Sage publications, 2017).
27 Charmaz, K. *Constructing grounded theory: A practical guide through qualitative analysis*. (Sage, 2006).
28 Glaser, B. G., Strauss, A. L. & Strutzel, E. The discovery of grounded theory; strategies for qualitative research. *Nursing research* **17**, 364 (1968).
29 Glaser, B. G. & Strauss, A. L. *Awareness of dying*. (Routledge, 2017).
30 Fassinger, R. E. Paradigms, praxis, problems, and promise: Grounded theory in counseling psychology research. *Journal of counseling psychology* **52**, 156 (2005).
31 Strauss, A. & Corbin, J. M. *Grounded theory in practice*. (Sage, 1997).
32 Weed, M. Research quality considerations for grounded theory research in sport & exercise psychology. *Psychology of sport and exercise* **10**, 502-510 (2009).
33 Lazenbatt, A. & Elliott, N. How to recognise a'quality'grounded theory research study. *Australian Journal of Advanced Nursing, The* **22**, 48 (2005).
34 Ali, N., May, S. & Grafton, K. A systematic review of grounded theory studies in physiotherapy. *Physiotherapy theory and practice*, 1-31 (2018).
35 Hutchison, A. J., Johnston, L. & Breckon, J. Grounded theory-based research within exercise psychology: A critical review. *Qualitative Research in Psychology* **8**, 247-272 (2011).

For peer review only - http://bmjopen.bmj.com/site/about/guidelines.xhtml

36 Douglas, D. Grounded theories of management: A methodological review. *Management Research News* **26**, 44-52 (2003).
37 da Silva Barreto, M., Garcia-Vivar, C. & Marcon, S. S. Methodological quality of Grounded Theory research with families living with chronic illness. *International journal of Africa nursing sciences* **8**, 14-22 (2018).
38 Charmaz, K. 'Discovering'chronic illness: using grounded theory. *Social science & medicine* **30**, 1161-1172 (1990).
39 Baker, C. & Stern, P. N. Finding meaning in chronic illness as the key to self-care. *Canadian Journal of Nursing Research Archive* **25** (1993).
40 Conrad, P. Qualitative research on chronic illness: a commentary on method and conceptual development. *Social Science & Medicine* **30**, 1257-1263 (1990).
41 Corbin, J. & Strauss, A. Managing chronic illness at home: three lines of work. *Qualitative sociology* **8**, 224-247 (1985).
42 Belgrave, L. L. & Charmaz, K. in *The Social Construction of Death* 34-51 (Springer, 2014).
43 Charmaz, K. Loss of self: a fundamental form of suffering in the chronically ill. *Sociology of health & illness* **5**, 168-195 (1983).
44 Charmaz, K. Grounded theory as an emergent method. *Handbook of emergent methods* **155**, 172 (2008).
45 Bernell, S. & Howard, S. W. Use your words carefully: what is a chronic disease? *Frontiers in public health* **4**, 159 (2016).
46 World Health Organization. Preventing chronic diseases: a vital investment. Report No. 9241563001, (World Health Organization, Public Health Agency of Canada, Canada. Public Health Agency of Canada, 2005).
47 Scinicariello, F., Carroll, Y., Eichwald, J., Decker, J. & Breysse, P. N. Association of Obesity with Hearing Impairment in Adolescents. *Scientific reports* **9**, 1877 (2019).
48 Blackwell, D. L., Lucas, J. W. & Clarke, T. C. Summary health statistics for US adults: national health interview survey, 2012. *Vital and health statistics. Series 10, Data from the National Health Survey*, 1-161 (2014).
49 Vos, T. *et al.* Global, regional, and national incidence, prevalence, and years lived with disability for 310 diseases and injuries, 1990–2015: a systematic analysis for the Global Burden of Disease Study 2015. *The Lancet* **388**, 1545-1602 (2016).
50 Meston, C. N. & Ng, S. L. A grounded theory primer for audiology. *Seminars in hearing* **33**, 135-146 (2012).
51 Timonen, V., Foley, G. & Conlon, C. Challenges when using grounded theory: A pragmatic introduction to doing GT research. *International Journal of Qualitative Methods* **17**, 1609406918758086 (2018).
52 Morse, J. M. *et al. Developing grounded theory: The second generation*. (Routledge, 2016).
53 Hussein, M. E., Hirst, S., Salyers, V. & Osuji, J. Using grounded theory as a method of inquiry: Advantages and disadvantages. *The Qualitative Report* **19**, 1-15 (2014).
54 Moher, D. *et al.* Preferred reporting items for systematic review and meta-analysis protocols (PRISMA-P) 2015 statement. *Systematic Reviews* **4**, doi:Artn 110.1186/2046-4053-4-1 (2015).

For peer review only - http://bmjopen.bmj.com/site/about/guidelines.xhtml

55 Shamseer, L. *et al.* Preferred reporting items for systematic review and meta-analysis protocols (PRISMA-P) 2015: elaboration and explanation. *Bmj-British Medical Journal* **349**, doi:ARTN g764710.1136/bmj.g7647 (2015).
56 Munn, Z., Stern, C., Aromataris, E., Lockwood, C. & Jordan, Z. What kind of systematic review should I conduct? A proposed typology and guidance for systematic reviewers in the medical and health sciences. *Bmc Medical Research Methodology* **18**, doi:ARTN 510.1186/s12874-017-0468-4 (2018).
57 Clarke, M., Oxman, A., Paulsen, E., Higgins, J. & Green, S. Appendix A: Guide to the contents of a Cochrane Methodology protocol and review. *Cochrane Handbook for Systematic Reviews of Interventions* (2009).
58 McIlroy, G. & Storbeck, C. Development of deaf identity: an ethnographic study. *J Deaf Stud Deaf Educ* **16**, 494-511, doi:10.1093/deafed/enr017 (2011).
59 Chapman, M. & Dammeyer, J. The Significance of Deaf Identity for Psychological Well-Being. *J Deaf Stud Deaf Educ*, doi:10.1093/deafed/enw078 (2016).
60 Leigh, I., Marcus, A., Dobosh, P. & Allen, T. Deaf/hearing cultural identity paradigms: modification of the deaf identity development scale. *J Deaf Stud Deaf Educ* **3**, 329-338, doi:10.1093/oxfordjournals.deafed.a014360 (1998).
61 British Society of Audiology,. Recommended procedure: pure-tone air-conduction and bone-conduction threshold audiometry with and without masking. United Kingdom; (2017).
62 Reviews, C. f. & Dissemination. *CRD's guidance for undertaking reviews in healthcare*. (York Publ. Services, 2009).
63 Misra, D. P. & Agarwal, V. Systematic Reviews: Challenges for their justification, related comprehensive searches, and implications. *J Korean Med Sci* **33** (2018).
64 Berthelsen, C. B., Grimshaw-Aagaard, S. L. S. & Hansen, C. Developing a Guideline for Reporting and Evaluating Grounded Theory Research Studies (GUREGT). *International Journal of Health Sciences* **6**, 64-76 (2018).
65 Thomas, J. & Harden, A. Methods for the thematic synthesis of qualitative research in systematic reviews. *Bmc Med Res Methodol* **8**, 45 (2008).
66 Ring, N. A., Ritchie, K., Mandava, L. & Jepson, R. A guide to synthesising qualitative research for researchers undertaking health technology assessments and systematic reviews. (2011).
67 Armstrong, D., Gosling, A., Weinman, J. & Marteau, T. The place of inter-rater reliability in qualitative research: an empirical study. *Sociology* **31**, 597-606 (1997).
68 McAlister, A. *et al.* in *ASEE Annual Conference & Exposition, Columbus, Ohio. https://peer.asee.org/28777.*
69 Lewin, S. *et al.* Using Qualitative Evidence in Decision Making for Health and Social Interventions: An Approach to Assess Confidence in Findings from Qualitative Evidence Syntheses (GRADE-CERQual). *Plos Med* **12**, doi:ARTN10.1371/journal.pmed.1001895 (2015).

For peer review only - http://bmjopen.bmj.com/site/about/guidelines.xhtml

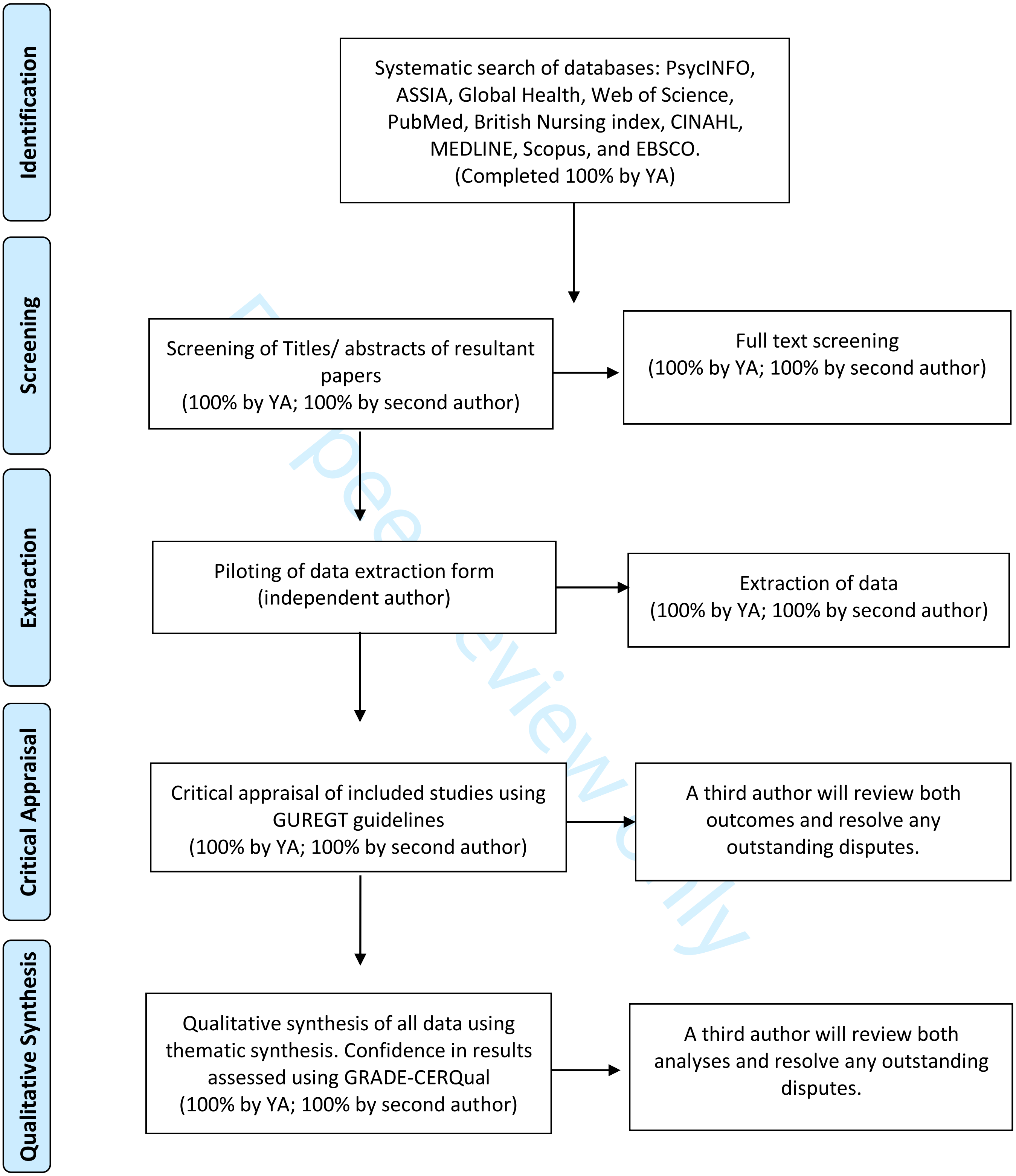

Figure 1. Flow Diagram of systematic review data screening, extraction and analysis processes.

PRISMA-P (Preferred Reporting Items for Systematic review and Meta-Analysis Protocols) 2015 checklist: recommended items to address in a systematic review protocol*

Section and topic	Item No	Checklist item	Completed? Yes/ No/ N/A	Page Numbers
**ADMINISTRATIVE INFORMATION**				
Title:				
Identification	1a	Identify the report as a protocol of a systematic review	Yes	1
Update	1b	If the protocol is for an update of a previous systematic review, identify as such	N/A	N/A
Registration	2	If registered, provide the name of the registry (such as PROSPERO) and registration number	Yes	3
Authors:				
Contact	3a	Provide name, institutional affiliation, e-mail address of all protocol authors; provide physical mailing address of corresponding author	Yes	1
Contributions	3b	Describe contributions of protocol authors and identify the guarantor of the review	Yes	17
Amendments	4	If the protocol represents an amendment of a previously completed or published protocol, identify as such and list changes; otherwise, state plan for documenting important protocol amendments	N/A	N/A
Support:				
Sources	5a	Indicate sources of financial or other support for the review	Yes	18
Sponsor	5b	Provide name for the review funder and/or sponsor	Yes	18
Role of sponsor or funder	5c	Describe roles of funder(s), sponsor(s), and/or institution(s), if any, in developing the protocol	Yes	18
**INTRODUCTION**				
Rationale	6	Describe the rationale for the review in the context of what is already known	Yes	3-7
Objectives	7	Provide an explicit statement of the question(s) the review will address with reference to participants, interventions, comparators, and outcomes (PICO)	Yes	7

For peer review only

**METHODS**				
Eligibility criteria	8	Specify the study characteristics (such as PICO, study design, setting, time frame) and report characteristics (such as years considered, language, publication status) to be used as criteria for eligibility for the review	Yes	8
Information sources	9	Describe all intended information sources (such as electronic databases, contact with study authors, trial registers or other grey literature sources) with planned dates of coverage	Yes	10
Search strategy	10	Present draft of search strategy to be used for at least one electronic database, including planned limits, such that it could be repeated	Yes	11-12
Study records:				
Data management	11a	Describe the mechanism(s) that will be used to manage records and data throughout the review	Yes	12
Selection process	11b	State the process that will be used for selecting studies (such as two independent reviewers) through each phase of the review (that is, screening, eligibility and inclusion in meta-analysis)	Yes	12
Data collection process	11c	Describe planned method of extracting data from reports (such as piloting forms, done independently, in duplicate), any processes for obtaining and confirming data from investigators	Yes	12-14
Data items	12	List and define all variables for which data will be sought (such as PICO items, funding sources), any pre-planned data assumptions and simplifications	Yes	13-14
Outcomes and prioritization	13	List and define all outcomes for which data will be sought, including prioritization of main and additional outcomes, with rationale	Yes	14-17
Risk of bias in individual studies	14	Describe anticipated methods for assessing risk of bias of individual studies, including whether this will be done at the outcome or study level, or both; state how this information will be used in data synthesis	Yes	15-16
Data synthesis	15a	Describe criteria under which study data will be qualitatively synthesised	Yes	14-15
	15b	If data are appropriate for qualitative synthesis, describe planned summary measures, methods of handling data and methods of combining data from studies, including any planned exploration of consistency (such as I^2, Kendall's τ)	Yes	14- 16
	15c	Describe any proposed additional analyses (such as sensitivity or subgroup analyses, meta-regression)	N/A	N/A

For peer review only

	15d	If quantitative synthesis is not appropriate, describe the type of summary planned	Yes	14-16
Meta-bias(es)	16	Specify any planned assessment of meta-bias(es) (such as publication bias across studies, selective reporting within studies)	Yes	15-16
Confidence in cumulative evidence	17	Describe how the strength of the body of evidence will be assessed (such as GRADE)	Yes	15-16

*** It is strongly recommended that this checklist be read in conjunction with the PRISMA-P Explanation and Elaboration (cite when available) for important clarification on the items. Amendments to a review protocol should be tracked and dated. The copyright for PRISMA-P (including checklist) is held by the PRISMA-P Group and is distributed under a Creative Commons Attribution Licence 4.0.**

From: Shamseer L, Moher D, Clarke M, Ghersi D, Liberati A, Petticrew M, Shekelle P, Stewart L, PRISMA-P Group. Preferred reporting items for systematic review and meta-analysis protocols (PRISMA-P) 2015: elaboration and explanation. BMJ. 2015 Jan 2;349(jan02 1):g7647.

For peer review only
